# Genome-wide association study meta-analysis of suicide death and suicidal behavior

**DOI:** 10.1038/s41380-022-01828-9

**Published:** 2022-10-17

**Authors:** Qingqin S. Li, Andrey A. Shabalin, Emily DiBlasi, Srihari Gopal, Carla M. Canuso, FinnGen, International Suicide Genetics Consortium, Aarno Palotie, Wayne C. Drevets, Anna R. Docherty, Hilary Coon

**Affiliations:** 1 Neuroscience, Janssen Research & Development, Titusville, NJ 08560 USA; 2 R&D Data Science, Janssen Research & Development, Titusville, NJ 08560 USA; 3 Huntsman Mental Health Institute, Department of Psychiatry, University of Utah School of Medicine, Salt Lake City, UT 84112 USA; 4 Institute for Molecular Medicine Finland, University of Helsinki, Helsinki, Finland; 5 Neuroscience, Janssen Research & Development, San Diego, CA 92121 USA; 6 Virginia Institute for Psychiatric & Behavioral Genetics, Virginia Commonwealth University School of Medicine, Richmond, VA USA; 7 Present Address: Regeneron Pharmaceuticals Inc, Tarrytown, NY 10591 USA

**Keywords:** Psychiatric disorders, Neuroscience, Genetics

## Abstract

Suicide is a worldwide health crisis. We aimed to identify genetic risk variants associated with suicide death and suicidal behavior. Meta-analysis for suicide death was performed using 3765 cases from Utah and matching 6572 controls of European ancestry. Meta-analysis for suicidal behavior using data across five cohorts (*n* = 8315 cases and 256,478 psychiatric or populational controls of European ancestry) was also performed. One locus in neuroligin 1 (*NLGN1*) passing the genome-wide significance threshold for suicide death was identified (top SNP rs73182688, with *p* = 5.48 × 10^−8^ before and *p* = 4.55 × 10^−8^ after mtCOJO analysis conditioning on MDD to remove genetic effects on suicide mediated by MDD). Conditioning on suicidal attempts did not significantly change the association strength (*p* = 6.02 × 10^−8^), suggesting suicide death specificity. *NLGN1* encodes a member of a family of neuronal cell surface proteins. Members of this family act as splice site-specific ligands for beta-neurexins and may be involved in synaptogenesis. The NRXN-NLGN pathway was previously implicated in suicide, autism, and schizophrenia. We additionally identified *ROBO2* and *ZNF28* associations with suicidal behavior in the meta-analysis across five cohorts in gene-based association analysis using MAGMA. Lastly, we replicated two loci including variants near *SOX5* and *LOC101928519* associated with suicidal attempts identified in the ISGC and MVP meta-analysis using the independent FinnGen samples. Suicide death and suicidal behavior showed positive genetic correlations with depression, schizophrenia, pain, and suicidal attempt, and negative genetic correlation with educational attainment. These correlations remained significant after conditioning on depression, suggesting pleiotropic effects among these traits. Bidirectional generalized summary-data-based Mendelian randomization analysis suggests that genetic risk for the suicidal attempt and suicide death are both bi-directionally causal for MDD.

## Introduction

Suicide is a worldwide public health crisis, accounting for close to 800,000 deaths per year. In 2019, suicide was the tenth leading cause of death [1], the second leading cause of death among individuals between the ages of 10 and 34, and the fourth leading cause of death among individuals between the ages of 35 and 54 in the United States [2]. The rate of suicidal behavior (SB) has climbed steadily over the past two decades [3–6]. Suicide outcomes encompass a range of behaviors. Non-suicidal self-injury (NSSI) is defined as the intentional destruction of body tissue without suicidal intent [7]. Suicidal attempt (SA), defined as nonfatal self-injurious behavior with the intent to die, has been estimated to occur over 20 times more frequently than suicide death (SD) and is a major source of disability, reduced quality of life, social and economic burden. SB includes both SD and SA. There are intricate relationships between trauma exposure, NSSI, suicidal ideation, SA, and SD with common genetic contributions to trauma exposure and self-injurious thoughts and behaviors [8]. The observations of a higher frequency of SD and SA in monozygotic twins compared to dizygotic twins among suicide twin survivors but not non-suicide twin survivors suggest a genetic contribution to SB [9–14]. Family studies suggest a significant genetic contribution with heritability ranging from 30 to 55% to suicidal thoughts and behaviors [15, 16], while SAs have heritability estimates of 17–45%, even after controlling for psychiatric disorders [17].

Psychiatric disorders are nevertheless a major risk factor for SB, and shared heritability has been demonstrated via polygenic risk score (PRS) analysis and/or genetic correlation, with the strongest genetic correlation with depression (*r*_*g*_ = 0.81) in the UK Biobank (UKB) [18, 19]. A prior SA is also a risk factor for SD and shared heritability is expected. However, based on conceptual and empirical differences (e.g., methods used, peak age, gender differences, frequency; CDC, 2016), nonfatal and fatal attempts are considered qualitatively distinct phenomena [20], suggesting genetic risk factors specific to SD may exist. Approximately two dozen genome-wide association studies (GWAS) using dichotomized traits of SA, SD, SB, and/or suicidal or self-harm ideation, or severity-based quantitative traits have been reported from both clinical and non-clinical population cohorts (civilians and military personnel) [18, 19, 21–42]. Most studies are from primarily European ancestry, but studies from other ancestries such as the Hispanic/Latino [27, 28, 41, 42], Asian [34, 41, 42], and African American [30, 41, 42] have started to emerge. Few genome-wide significant loci have been identified, and to date, replication has occurred for genome-wide significant association signals from chromosome 7, and variants near *LDHB* (European) and *FAH* (African American) [26, 30]. The largest meta-analysis to date between International Suicide Genetics Consortium (ISGC) and MVP identified 12 genome-wide significant loci [42]. Significant chip-based SNP heritability was estimated from several GWAS as well, with a heritability of 3.5% in the UKB (*p* = 7.12 × 10^−4^) [24], 4.6% in a Danish cohort (heritability was reduced to 1.9% after adjusting for mental disorders) [25], and 6.8% in the ISGC meta-analysis [26]. Gene-by-environment genome-wide interaction study was reported identifying PTSD as the main environment driver and a replicated male-associated genome-wide signal near *CWC22* [43]. In addition, the contribution of rare variants by whole-exome sequencing was evaluated in UKB for the ever-attempted suicide and suicidal ideation phenotypes, and no significant finding was reported using the study-wide significance threshold of 2.18 × 10^−11^ [44]. Lastly, an increased global copy number variation (CNV) rate was reported for SA cases and a common CNV verified by qPCR assay near *ZNF33B* was reported in a small MDD cohort [45].

Since the recently published genomic analysis of SD data from a large population-ascertained cohort from Utah [22], we have genotyped another ~1200 samples from the original cohort and matched the cases with controls genotyped using a matching array platform. We additionally added three cohorts of subjects with a lifetime history of suicide attempts (FinnGen cohort and two Janssen clinical trial samples). We aim to further interrogate the genetic basis of suicidal death and SB and further understand the relationship between SA and suicidal death. We also would like to interrogate SD-specific and SA-specific genetic risk factors by performing conditional analysis on the most correlated psychiatric condition (depression), and SA (for the SD phenotype only). Lastly, we would like to further dissect the genetic architecture of SD, SA, and SB by understanding the shared genetic components and causal relationship between these traits and psychiatric/non-psychiatric traits.

## Subjects and methods

### Cohorts and sample ascertainment

A total of five cohorts were included in this study (Supplementary Method S1). Cohorts 1 and 2 consist of SD cases from the University of Utah [22] and matching controls from Janssen Research & Development, LLC and dbGaP. The Utah case samples were genotyped in three waves to date. Wave 1 and 2 samples were included in a previous report [22] except that the cases were matched to different sets of controls (Generation Scotland samples genotyped using OmniExpress and UK10K samples with whole-genome sequencing data). Compared to the previous SD GWAS (3413 cases) [22], a total of 3765 cases were included between cohorts 1 and 2, among which 2832 are common while 581 were unique in the previous SD GWAS analysis and 933 cases were unique in this study.

Cohorts 3 and 4 consist of suicide attempt cases and controls of European ancestry and were drawn from 12 clinical trial samples (NCT00044681, NCT00397033, NCT00412373, NCT00334126, NCT01193153, NCT02497287, NCT02422186, NCT01627782, NCT00253162, NCT00257075, NCT01515423, and NCT01529515) conducted by Janssen Research & Development, LLC. A subset of samples from cohorts 3 and 4 were included in a previous GWAS [26]. All Janssen clinical studies were approved by the appropriate institutional review boards or ethnic committees and have followed the principles outlined in the Declaration of Helsinki for all human investigations. In addition, informed consent has been obtained from the study participants involved.

Cohort 5 consists of SA cases and controls from FinnGen (https://www.finngen.fi/en/about). The SA analysis using FinnGen data release 6 (R6) from the FinnGen Study included 4098 individuals with SA history, defined as the presence of SA International Classification of Diseases codes, and 247,898 individuals without the relevant codes. The diagnosis codes used to define SA are provided in Supplementary Table S1. The detailed descriptions of these cohorts are available in Supplementary Method S1.

Cohorts 1 and 2 were used for SD GWAS, while all five cohorts were used for SB GWAS.

### Genotyping and quality control

SD cases were genotyped using the Infinium PsychArray platform (Illumina, Inc., San Diego, CA, Supplementary Method S2). Janssen’s SA cases and control samples were genotyped using either PsychArray, Human1M-Duo, or HumanOmni5Exome (Illumina, Inc., San Diego, CA). QC was performed initially by a local QC pipeline by genotyping batch, while the combined data were QC’ed again using RICOPILI [46] pipeline. Additional details on QC, principal component analysis (PCA), case–control matching, and imputation can be found in Supplementary Method S3.

#### Genome-wide association analysis

For the Utah SD association analysis, a linear mixed model (LMM) algorithm was used to test the association between variants and SD. For SD cohort 1, GWAS were performed using GEMMA [47], a computationally efficient yet suitable for smaller sample size, and an open-source LMM algorithm for GWAS modeling population stratification remaining after PCA by use of genomic relatedness matrices. For SD cohort 2, GWAS was performed using BOLT-LMM [48] that implements an extremely efficient Bayesian mixed-model analysis and is suitable in large cohorts (requiring N to be at least 5000). For the FinnGen cohort, GWAS was performed using the standard FinnGen pipeline that implements the LMM algorithm using SAIGE [49] that efficiently controls for case–control imbalance and sample relatedness. For the two Janssen SA cohorts, the standard logistic regression using PLINK as implemented in the RICOPILI pipeline was used. Meta-analysis was performed using METAL [50] (as implemented in the RICOPILI pipeline) for the two SD cohorts to identify genetic variants associated with SD and across all five cohorts to identify genetic variants associated with SB. Conventional genome-wide significance threshold 5 × 10^−8^ is used to declare study-wide significance. A list of variants with unadjusted *p* value less than 5 × 10^−6^ is also reported.

It is well known that substantial genetic liability is shared across psychiatric traits. To identify putative SD-specific genetic associations, multi-trait conditional and joint analysis (mtCOJO) [51] was further used to adjust using GWAS summary statistics for the effects of genetically correlated traits (MDD and SA). For MDD and SAs, the GWAS summary statistics from Howard et al. [52], without 23andMe cohort and Mullin et al. [26] without SD cohort (Utah wave 1 and 2 data and two other cohorts with predominant SD cases) were used (Supplementary Method S4), respectively. Likewise, to identify SB-specific genetic associations, mtCOJO was also used to adjust for the effects of MDD to identify putative SB-specific genetic associations. mtCOJO analyses were performed using Genome-wide Complex Trait Analysis version 1.93.2 beta [53].

#### Variant annotation and multi-marker analysis of genomic annotation (MAGMA) gene- and gene-set-based analysis

Variant clumping to identify independent genomic locus and annotation was performed using FUMA [54]. In addition to single-marker-based GWAS, gene-based analyses followed by pathway enrichment analysis were computed using MAGMA [55] based on GWAS meta-analysis summary statistics. SNPs were mapped to 18,927 protein-coding genes. Genome-wide significance was defined at *p* = 0.05/18,927 = 2.64 × 10^−6^. The MAGMA analyses were performed using FUMA [54].

### Replication of genome-wide significant loci from ISGC and Million Veteran Program suicidal attempt meta-analysis

Among the five cohorts used in this study, the FinnGen cohort was completely independent of the cohorts included in the ISGC and Million Veteran Program meta-analysis [42]. We used the results from the FinnGen cohort to replicate the 12 genome-wide significant loci reported in the meta-analysis between ISGC and MVP cohorts [42]. SNPs with an association *p* value less than 0.05/12~0.00417 were considered replicated. Other cross-references/replication attempts of published results are also described in Supplementary Method S5.

#### Polygenic risk score association with suicide death

PRSs derived from 92 summary statistics (for non-unique traits) were tested for association with SD for cohort 1 and cohort 2, respectively, using PRSice-2 [56] with P_T_ fixed at 1. Traits for calculating PRS included both psychiatric and somatic comorbidities, personality traits, and lifestyle factors. A full list of PRS derived is available in Supplementary Table S2 and Supplementary Method S6. Association *p* value less than 0.05/92~0.00054 was considered significant. The association results between cohort 1 and cohort 2 were compared for consistency. The results were also compared to the published PRS prediction or genetic correlation analysis results.

#### SNP heritability and genetic correlation estimations

The phenotypic variance explained by variants (both genotyped and imputed, mostly SNPs) (*h*^2^_SNP_) for each of the phenotype groups was estimated using association statistics as implemented in linkage disequilibrium (LD) Score regression [57]. Genetic correlations between a smaller list of selected traits (psychiatric and non-psychiatric as described in Supplementary Method S7) and SD and SB both before and after mtCOJO adjustments were also evaluated using LD Score regression. ISGC SA [26] (Supplementary Method S4) was also included as a reference for the calculation of genetic correlation. Details of summary statistics used for these traits together with population prevalence rate assumptions are available in Supplementary Table S3. Genetic correlation with a *p* value less than 0.05/18~0.0028 was considered significant as we accounted for the number of non-suicide traits for multiple testing corrections.

#### Generalized summary-data-based Mendelian randomization (GSMR)

GSMR [51] is a method to test for a putative causal association between a risk factor and a disease using summary-level data from GWAS. In this study, we test the relationship between MDD and SD/SA as well as the relationship between SA and SD.

## Results

For the SD cohorts from the University of Utah and the SA cohort from FinnGen, psychiatry conditions were more prevalent among the cases as expected (Table 1).

**Table 1:** Summary of cohorts used in this study.

	Cohorts 1 and 2	Cohort 3 (PsychArray)		Cohort 4 (Omni1M^c^)		Cohort 5	
Affection status	Cases^a^	Cases	Controls^b^	Cases	Controls^b^	Cases	Controls^b^
Case phenotype	Suicide death	Suicidal attempts		Suicidal attempts		Suicidal attempts	
*N*	3765	183	1199	268	780	4098	247,898
Cases with linked demographic data, *N*	3426	183	1199	268	780	4098	247,898
Cases with linked demographic data and EHR, *N*	2981	N/A	N/A	N/A	N/A	4098	247,898
*Demographic information*							
Female, *n* (%)	743 (21.7)	95 (51.9)	612 (51.0)	142 (53.0)	435 (55.8)	3525 (86.0)	138,666 (55.9)
Age, mean (SD)	41.0 (17.2)	47.1 (13.0)	44.3 (14.2)	41.2 (11.8)	43.6 (12.5)	56.6 (17.8)	59.6 (17.7)
Clinical characteristics, *n* (%)							
Bipolar disorder	321 (10.8)			91 (34.0)	223 (28.6)	257 (6.3)	3428 (1.4)
Broad bipolar spectrum illness	450 (15.1)					295 (7.2)	4130 (1.7)
MDD	927 (31.1)	87 (47.3)	492 (41.0)	93 (34.7)	355 (45.5)	1020 (24.9)	20,691 (8.3)
Broad depression	1568 (52.6)					1141 (27.8)	24,379 (9.8)
Anxiety	747 (25.1)					844 (20.6)	17,576 (7.1)
Schizophrenia	117 (3.9)	53 (28.8)	579 (48.3)	20 (7.5)	87 (11.2)	222 (5.4)	2634 (1.1)
Psychosis	295 (9.9)					673 (16.4)	13,185 (5.3)
Pain	1636 (54.9)					40 (1.0)	1012 (0.4)
Sleep problems	822 (27.6)					328 (8.0)	9312 (3.8)
SUD	1513 (50.8)					739 (18.0)	13,895 (5.6)
SLE	283 (9.5)					–	–
Suicidal ideation	560 (18.8)					–	–
Adult maltreatment	7 (0.2)					–	–
Childhood maltreatment	15 (0.5)					–	–
Any mental health	2142 (71.9)					2378 (58.0)	73,576 (29.7)
Diabetes	278 (9.3)						
Asthma	359 (12.0)						
Schizoaffective disorder		43 (23.5)	128 (10.7)	64 (23.9)	115 4.7)		

Specifically, cohorts 1 and 2 are used for suicide death (SD) GWAS meta-analysis, and cohorts 1–5 are used for suicidal behavior (SB) GWAS meta-analysis.

^a^The controls for cohort 1 are Coriell controls screened for psychiatric medical and family history (*n* = 804), while the controls for cohort 2 are IAMDGC controls not screened for suicide attempt (*n* = 5768).

^b^The controls for cohorts 3 and 4 are psychiatric controls within the same studies.

^c^Using the overlapping markers between Omni1M-Duo and Omni5M.

### Genome-wide association (SNP-level and gene-level associations)

**Fig. 1:**
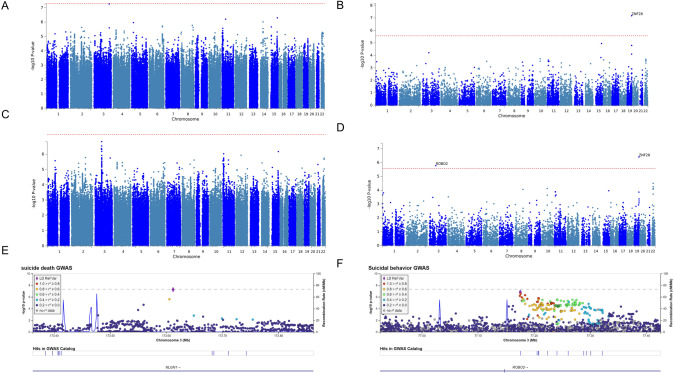
Genome-wide significant association signals. Manhattan plots for suicide death GWAS meta-analysis: SNP-level (**A**), gene-level (**B**); and suicidal behavior GWAS meta-analysis: SNP-level (**C**), gene-level (**D**). Regional plot for *NLGN1* (**E**); regional plot for *ROBO2* (**F**). The dotted line indicates a genome-wide significance threshold of 5 × 10^–8^. For the regional association plot generated by LocusZoom [95], SNPs in genomic risk loci are color-coded as a function of their *r*^2^ to the index SNP in the locus, as follows: red (*r*^2^ > 0.8), orange (*r*^2^ > 0.6), green (*r*^2^ > 0.4) and light blue (*r*^2^ > 0.2). SNPs that are not in LD with the index SNP (with *r*^2^ ≤ 0.2) are dark blue, while SNPs with missing LD information are shown in gray.

For the expanded SD GWAS meta-analysis using 3765 cases and 6572 populational controls genotyped (Table 1 and Supplementary Method S1), one locus in neuroligin 1 (*NLGN1*) passing genome-wide significance threshold for SD meta-analysis compared to the general population was identified (top SNP rs73182688, with *p* = 5.48 × 10^−8^ before and *p* = 4.55 × 10^−8^ after mtCOJO analysis conditioning on MDD summary statistics (Table 2); Manhattan plots in Fig. 1A and Supplementary Fig. S3A, QQ-plots in Supplementary Fig. S2A, B). Conditioning on SA summary statistics (*p* = 6.02 × 10^−8^, Manhattan plot in Supplementary Fig. S3B, QQ-plot in Supplementary Fig. S2C) or both MDD and SA summary statistics (*p* = 4.70 × 10^−8^) did not significantly change the association strength, suggesting that this is an SD-specific genetic risk locus. *NLGN1* encodes a member of a family of neuronal cell surface proteins. Members of this family act as splice site-specific ligands for beta-neurexins and the NLGN1 protein is involved in the formation and remodeling of central nervous system synapses. Additional variants associated with SD with suggestive association *p* value less than 5 × 10^−6^ and the annotated genes based on positional, eQTL, or chromatin interaction mapping are listed in Supplementary Tables S4 and S5, respectively. In addition, our study replicated the genome-wide significant finding in rs116955121 [19] that was associated with suicidal ideation and attempt in UKB (*p* = 0.0003 in our SD GWAS meta-analysis, Supplementary Table S6). Additional replication attempts were described in Supplementary Text S1 and Supplementary Tables S7 and S8. Among the 22 implicated genes associated with SD, 6 were significantly differentially expressed in postmortem brain samples of schizophrenia, autism, and/or bipolar disorder (*p* value less than 0.05/22~0.0023) based on the analysis from the PsychENCODE Consortium (Supplementary Table S9). Of particular interest, *EIF4G2* were consistently downregulated in both schizophrenia (*p* = 1.88 × 10^−5^) and bipolar disorder (*p* = 0.001).

**Table 2:** Top genome-wide association signals.

Analysis	CHR	SNP	BP	A1	A2	FRQ_A	FRQ_U	INFO	OR	SE	*p*	Direction	*N*_ca_	*N*_co_
Suicide death	3	**rs73182688**	173,612,201	A	G	0.885	0.863	0.603	1.06855	0.0122	5.48E–08	++	3765	6572
Suicide death | MDD											4.55E–08			
Suicide death | attempt											6.02E–08			
Suicide death | MDD and attempt											4.70E–08			
Suicidal behavior	3	rs7649370	77,175,484	A	G	0.645	0.677	0.989	1.03448	0.0065	1.51E–07	+++++	8315	256,478
Suicidal behavior | MDD											2.89E–07			
Suicidal behavior	3	rs6782116	77,176,032	C	T	0.651	0.68	0.986	1.03386	0.0065	2.92E–07	–––––	8315	256,478
Suicidal behavior | MDD											4.38E–07			

A1: Allele 1 (odds ratios calculated with respect to this allele), A2: Allele 2, INFO: quality of imputation for the SNP.

*BP* base-pair position in reference to hg19, *CHR* chromosome, *FRQ_A* minor allele frequency (A1) in cases, *FRQ_U* minor allele frequency (A1) in controls, *OR* odds ratio, *SE* standard error, *SNP* single-nucleotide polymorphism.

SNP in bold reached genome-wide significance threshold in either the main analysis or mtCOJO conditional analysis.

Gene-based association using MAGMA additionally identified *ZNF28* to be associated with SD (Manhattan plot in Fig. 1B, QQ-plot in Supplementary Fig. S2D, regional plot in Fig. 1E), and the association did not weaken when adjusting for correlated traits including MDD and SA (Manhattan plots in Supplementary Fig. S3C, D, QQ-plots in Supplementary Fig. S2E, F). Additional regional plots for suggestive association signals from the SD GWAS are available in Supplementary Fig. S4. The top 10 genes with suggestive evidence associated with SD are also listed in Supplementary Table S10.

No variant associated with SB passed the genome-wide significance threshold in the meta-analysis across five cohorts (*n* = 8315 cases and 256,478 psychiatric or populational controls, Table 1) before (Manhattan plot in Fig. 1C, QQ-plot in Supplementary Fig. S2G) and after (Manhattan plot in Supplementary Fig. S3E, QQ-plot in Supplementary Fig. S2H) applying the mtCOJO adjustment for MDD. Additional variants associated with SB with suggestive association *p* value less than 5 × 10^−6^ and the corresponding implicated genes are listed in Supplementary Tables S11 and S12 with regional plots available in Supplementary Fig. S5. Genes with differential gene expression evidence from PsychENCODE are also provided in Supplementary Table S13. SB “replication” attempt of ISGC results is described in Supplementary Text S1 and Supplementary Tables S14 and S15. Among the 12 genome-wide significant loci identified from the ISGC and Million Veteran Program SA meta-analysis, two associations with SA (equivalent to our SB endpoint in this study) were replicated accounting for 12 independent tests in the FinnGen cohort with consistent directionality (rs17485141 [*p* = 0.003, OR = 0.92] near SRY-box transcription factor 5 (*SOX5*) and rs62404522 near *LOC101928519* [*p* = 0.001, OR = 1.12]. There were two other associations trended toward nominal associations (rs2503185 [*p* = 0.08] near *PDE4B* and rs7131627 near *DRD2* [*p* = 0.08]) with consistent directionality. The other nominal association (rs3791129 [*p* = 0.04] near *SLC6A9*) was in opposite directionality (Table 3).

**Table 3:** Replication results for genome-wide significant loci identified in the ISGC and MVP meta-analysis.

CHR	SNP	BP	A1	A2	FRQ_A_4098	FRQ_U_247898	INFO	OR	SE	*p*	Nearest gene (distance to index SNP in kb)
1	rs3791129	44,480,093	A	G	0.7582	0.7503	0.99	1.06	0.03	0.041	*SLC6A9* (0.0)
1	rs2503185	66,461,401	G	A	0.4231	0.4347	1	0.96	0.02	0.078	*PDE4B* (0.0)
11	rs7131627	113,299,829	A	G	0.4986	0.5107	1	0.96	0.02	0.075	*DRD2* (0.0)
12	rs17485141	24,213,634	T	C	0.2325	0.248	1	0.92	0.03	**0.003**	*SOX5* (0.0)
13	rs9525171	96,908,223	G	C	0.4996	0.5072	1	0.97	0.02	0.259	*HS6ST3* (0.0)
14	rs850261	57,346,423	G	A	0.4344	0.4274	1	1.04	0.02	0.114	OTX2-AS1 (0.0)
15	rs17514846	91,416,550	A	C	0.3905	0.3936	1	0.99	0.02	0.59	*FURIN* (0.0)
22	rs2284000	37,053,338	G	C	0.2922	0.2902	0.99	1	0.03	0.853	*CACNG2* (0.0)
3	rs7649709	173,129,819	A	C	0.3291	0.3269	0.96	1	0.02	0.965	*NLGN1* (0.0)
6	rs62404522	19,307,114	C	T	0.1416	0.1301	1	1.12	0.03	**0.001**	*LOC101928519* (–76.4)
6	rs35869525	26,946,687	T	C	0.04866	0.05079	1	0.98	0.05	0.642	MHC
7	rs62474683	115,020,725	G	A	0.4203	0.4283	1	0.97	0.02	0.172	*LINC01392* (–149.3)

A1: Allele 1 (odds ratios calculated with respect to this allele), A2: Allele 2, INFO: quality of imputation for the SNP.

*BP* base-pair position in reference to hg19, *CHR* chromosome, *FRQ_A* minor allele frequency (A1) in cases, *FRQ_U* minor allele frequency (A1) in controls, *OR* odds ratio, *SE* standard error, *SNP* single-nucleotide polymorphism.

*P*-values in bold signify replication with consistent directional effects.

Gene-based association using MAGMA additionally identified *ROBO2* and *ZNF28* passing study-wide significance as being associated with SB (Manhattan plot in Fig. 1D, QQ-plot in Supplementary Fig. S2I), this association did not weaken significantly when adjusting for MDD using mtCOJO, suggesting that the association is SB-specific (Manhattan plot in Supplementary Fig. S3F, QQ-plot in Supplementary Fig. S2J). It is noteworthy that the study-wide significant gene-based association for *ROBO2* corresponds to the most significant suggestive association in the SNP-based meta-analysis. *ROBO2* encodes a transmembrane receptor for the slit homolog 2 protein and functions in axon guidance and cell migration. The top 10 genes with suggestive evidence associated with SB are listed in Supplementary Table S10. There are also a few genes with suggestive evidence in multiple analyses, such as *ROBO2*, which had suggestive evidence associated with SD (*p* = 6.21 × 10^−5^ before adjusting with depression, *p* = 1.58 × 10^−4^ after adjusting with depression, *p* = 1.46 × 10^−4^ after conditioning on SA). The same is true for a few other brain-expressed genes such as *LIMK2*, *NRBF2*, *NRG1*, and *ZNF710* (Supplementary Table S10). Among the 18 implicated genes across all traits from the gene-based analysis, three of them were significantly differentially expressed in postmortem brain samples of schizophrenia, autism, and/or bipolar disorder (*p* value less than 0.05/18~0.00278) based on the analysis from PsychENCODE Consortium (Supplementary Table S16). Of particular interest, *LIMK2* were consistently upregulated in ASD (*p* = 0.0003), schizophrenia (*p* = 3.18 × 10^−9^), and bipolar disorder (*p* = 0.0003).

### MAGMA gene-set analysis

MAGMA gene-set enrichment analysis revealed that MHC class Ib receptor activity pathway was enriched among variants associated with SD (*p* = 1.82 × 10^−7^, Bonferroni-corrected *p* value = 2.82 × 10^–3^). This gene-set enrichment was driven by gene-based association results from leukocyte immunoglobulin-like receptor B1 (*LILRB1*, *p* = 0.003) in chromosome 19 and *CD160* (*p* = 0.07) in chromosome 1. There was suggestive enrichment for immune-related pathways, such as natural killer cell cytokine production (*p* = 7.68 × 10^–6^), positive regulation of interferon-gamma secretion (*p* = 3.39 × 10^–5^), immunological memory processing (*p* = 5.76 × 10^–5^), and negative regulation of viral-induced cytoplasmic pattern recognition receptor signaling pathway (*p* = 9.10 × 10^–5^) (Supplementary Table S17).

### Polygenic risk score association with suicide death

**Fig. 2:**
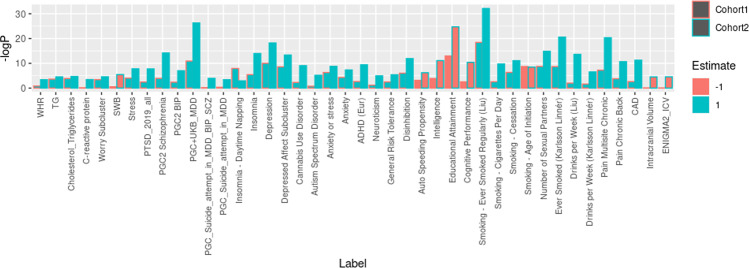
Polygenic risk score association with suicide death. *P* values plotted are association *p* value for the respective PRS and suicide death status in cohorts 1 and 2, respectively. Bar plots filled in red denote a negative association coefficient, while green ones denote a positive association coefficient. ADHD attention-deficit/hyperactivity disorder, ASD autism spectrum disorder, BIP bipolar disorder, CAD coronary artery disease, ICV intracranial volume, MDD major depressive disorder, PTSD posttraumatic stress disorder, SCZ schizophrenia, SWB subjective well-being, TG triglycerides, WHR waist-to-hip ratio.

Among the 92 PRSs derived from psychiatric, personality, somatic comorbidity, and lifestyle traits, 22 and 41 were associated with SD status in cohort 1 and cohort 2, respectively (Fig. 2), among which 21 were common. In both cohorts, PRSs derived from depression, anxiety, stress, insomnia, schizophrenia, and pain were positively associated while smoking-related traits and education attainment/intelligence were negatively associated with SD. Cohort 2 was much larger in sample size and revealed additional positive PRS associations derived from bipolar disorder, PTSD, general anxiety disorder (GAD), ASD, ADHD, substance use disorder (SUD), neuroticism, cholesterol/triglycerides, and negative associations derived from subjective well-being, intracranial volume, and cognitive performance. Many of these traits including depression, anxiety, pain, neuroticism, schizophrenia, bipolar disorder, and PTSD were previously reported to be genetically correlated with suicidality [19, 26]. A complete list of associations passing multiple test corrections is available in Supplementary Table S18.

### Genetic heritability of suicidal death, suicidal attempt, and suicidal behavior and genetic correlation with other traits

Total Liability scale *h*^*2*^_SNP_ for SD (from this study) and SA (from ISGC) was 5.02% and 5.45%, respectively. Conditioning on MDD, SA, or both reduced the *h*^*2*^_SNP_ for SD to 3.93%, 4.6%, and 3.65%, respectively. Total Liability scale *h*^*2*^_SNP_ for SB (from this study) was 2.98%, while conditioning on MDD reduced it to 2.19%.

**Fig. 3:**
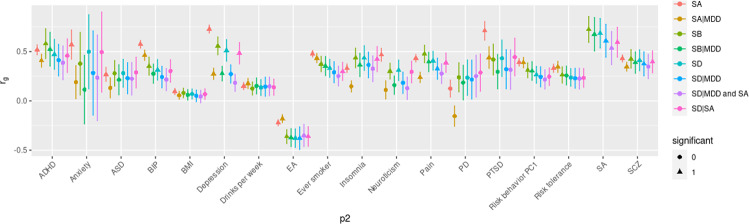
Genetic correlation between suicide death, suicidal attempt, and suicidal behavior (p1), and selected psychiatric/non-psychiatry traits (p2). Triangle points indicate genetic correlations that passed the Bonferroni-corrected significance threshold (*p* < 2.78 × 10^–3^). Error bars represent the standard error. ADHD attention-deficit/hyperactivity disorder, ASD autism spectrum disorder, BIP bipolar disorder, MDD major depressive disorder, PTSD posttraumatic stress disorder, SA suicidal attempt, SB suicidal behavior, SB|MDD SB results after conditioning on MDD, SCZ schizophrenia, SD suicide death, SD|MDD SD results after conditioning on MDD, SD|SA SD results after conditioning on SA, SD|MDD and SA SD results after conditioning on MDD and SA.

The genetic correlation between SD and SA was 0.69 (*p* = 4.16 × 10^–6^). Both traits were correlated with psychiatric trait depression (Fig. 3 and Supplementary Table S18). Specially, the genetic correlation between SD and MDD was 0.51 (SE = 0.11, *p* = 2.92 × 10^–6^), while the genetic correlation between SA (ISGC excluding SD cohorts) and MDD was 0.73 (SE = 0.04, *p* = 1.41 × 10–^72^). Likewise, the genetic correlation between SB and MDD was 0.56 (SE = 0.09, *p* = 6.84 × 10^–10^). After conditioning on depression, the correlations with depression were reduced (for SD|MDD, *r*_*g*_ = 0.27, *p* = 0.003; for SB|MDD, *r*_*g*_ = 0.28, *p* = 0.0002).

SA (based on ISGC summary statistics excluding SD cohorts) was used as a positive control in this study and the detailed results are provided in Supplementary Text S1. SB was correlated with SA (*r*_*g*_ = 0.72, *p* = 3.09 × 10^–8^), pain (*r*_*g*_ = 0.48, *p* = 8.15 × 10^–8^), educational attainment (*r*_*g*_ = –0.36, *p* = 2.51 × 10^–7^), ever smoker (*r*_*g*_ = 0.37, *p* = 1.41 × 10^–6^), schizophrenia (*r*_*g*_ = 0.43, *p* = 6.06 × 10^–6^), and insomnia (*r*_*g*_ = 0.44, *p* = 1.28 × 10^–5^). SD was also associated with pain and educational attainment. To examine whether these genetic correlations were mediated by depression, r_g_ was estimated with the same traits using the SB|MDD, and SD|MDD results. For SB and SD, genetic correlations with ASD, anxiety, PTSD were not significant before conditioning, while the genetic correlations with ADHD, insomnia (for SD|MDD only), risk tolerance (for SB|MDD only), bipolar disorder, and neuroticism (for both SB|MDD, and SD|MDD) became nonsignificant after conditioning. After conditioning on both MDD and SA, most of the genetic correlations with SD were nonsignificant except for the negative correlation with EA (Fig. 3 and Supplementary Table S19).

### Generalized summary-data-based Mendelian randomization (GSMR)

Bidirectional GSMR analysis suggests that the genetic risk for the SA and SD are both bidirectional causal with the genetic risk for MDD (Supplementary Fig. S6). Specifically, we found significant bidirectional causal relationships in SNP effect sizes for MDD loci in the genetic risk for SAs (*p*_GSMR_ = 8.30 × 10^–63^) and SA loci in MDD (*p*_GSMR_ = 2.65 × 10^–9^). In addition, we also found significant bidirectional causal relationships in SNP effect sizes for MDD loci in the genetic risk for SD (*p*_GSMR_ = 8.2 × 10^–5^) and SD loci in MDD (*p*_GSMR_ = 0.05).

## Discussion

Using a total of 3765 SD cases and 6572 populational controls, we identified one locus in neuroligin 1 (*NLGN1*) with genome-wide significance. The top SNP is rs73182688, with *p* = 5.48 × 10^–8^ before and *p* = 4.55 × 10^–8^ after mtCOJO analysis conditioning on depression; Howard et al., using summary statistics without the 23andMe cohort). Conditioning on the SA (ISGC summary statistics [26] without Utah and two other SD cohorts) did not significantly change the association strength (*p* = 6.02 × 10^–8^), suggesting this is a locus with SD specificity. Gene-based association using MAGMA additionally identified *ROBO2* and *ZNF28* as being associated with SB in the meta-analysis across five cohorts consisting of 8315 cases and 256,478 psychiatric or populational controls, among which *ZNF28* was also associated with SD. The gene-set enrichment analysis identified the MHC Class Ib receptor activity pathway as being significantly associated with SD. Using a completely independent sample set from FinnGen, we replicated two genome-wide significant findings from the ISGC and MVP meta-analysis including variants near *SOX5*.

Among the genes near the replicated variants associated with SA, *SOX5* was previously associated with schizophrenia, depression, neuroticism, chronotype, chronic back pain, C-reactive protein levels, cortical thickness, and surface area [52, 58–63], and is among a panel of genes contributing to the bidirectional causal effect of neuroticism on MDD [64]. The variant associated with depression (rs78337797) and the variant associated with SA (rs17485141) are in weak LD with each other (*r*^2^ = 0.13, *D*’ = 0.75), suggesting allelic heterogeneity and pleotropic effect of this locus. *SOX5* encodes a transcription factor important for embryogenesis and cell fate determination with the expression level highest during fetal development (Supplementary Fig. S7). A full list of reported genome-wide significant associations annotated to *SOX5* is provided in Supplementary Table S20.

Among the genes associated with SD, *NLGN1* encodes a member of a family of postsynaptic neuronal cell surface proteins. Members of this family act as splice site-specific ligands for presynaptic β-neurexins and are involved in the formation and remodeling of central nervous system synapses [65, 66]. Another variant in *NLGN1* (weak LD with the variant reported herein) is associated with SA in the ISGC and MVP meta-analysis, suggesting allelic heterogeneity in this gene. Neurexin 1 variants were previously implicated as risk factors for SD [67, 68]. The top associated variant rs73182688 in *NLGN1* in this study is nominally associated with BMI (*p* = 0.0006), depression (*p* = 0.004 in FinnGen R5), and personality disorder (*p* = 0.004 in FinnGen R5) (Supplementary Table S21). Other variants (SNVs and CNVs) in *NLGN1* and/or other family members *NLGN3* and *NLGN4* were previously associated with PTSD, autism, obsessive-compulsive disorder, and depression [69–75]. The rs6779753 variant in *NLGN1* associated with PTSD as well as with the intermediate phenotypes of higher startle response and greater hemodynamic responses (assessed using functional MRI) of the amygdala and orbitofrontal cortex to fearful face stimuli was not in LD with the variant identified herein. In our study, rs6779753 was only suggestively associated with SD (*p* = 0.06). *NLGN1* was also implicated in a preclinical model of depression [76]. In addition, presynaptic neurexins and cytoplasm partners such as SHANK also have been implicated in autism, schizophrenia, and mental retardation [73, 77–83]. Overall, there is substantial genetic evidence on the NRXN-NLGN pathway in suicide and other psychiatric conditions.

*ROBO2* and *ZNF28* were associated with SB in our study. Variants in *ROBO2* were previously reported to be associated with circadian phenotypes such as self-identification as a “morning person” and chronotype [62], smoking initiation [84], and highest math classes taken [85], all reaching genome-wide significance threshold. These genome-wide significant variants are in LD with the top variant rs7649370 from this study (*r*^2^ > 0.86). *ROBO2* was also implicated in schizophrenia, and psychopathic tendencies, although a subsequent study replicated the emotionally reactive, impulsive aspects of conduct disorder, but not the concurrent risk for psychopathy [86–88]. *ZNF28*, on the other hand, was not previously associated with psychiatric disorders. A few genes with gene-level suggestive association evidence across multiple analyses were discussed in Supplementary Text S3.

Consistent with the previous SD GWAS [22], elevated PRS for disinhibition, MDD, schizophrenia, and ASD in SD cases were observed in this study. The previously reported PRS associations for child IQ (*p* = 0.03 in cohort 1 and *p* = 0.95 in cohort 2) and loneliness (*p* = 0.04 and *p* = 0.06) were nominal in this study. This study however uncovered additional evidence of elevated PRS in anxiety, insomnia, stress, smoking, alcohol use, and pain among SD cases, consistent with epidemiology evidence, known risk factors, and warning signs noted by several suicides- or health-focused organizations, a previous meta-analysis on predictors of suicidal thought and behaviors, and/or previous reported genetic correlations [20, 26, 89–92]. Reduced PRS for education attainment and intelligence was associated with SD. Cohort 2 revealed additional positive PRS associations for bipolar disorder, PTSD, GAD, ASD, ADHD, SUD, neuroticism, cholesterol/triglycerides, and negative associations for subjective well-being, intracranial volume, and cognitive performance. While the last SD GWAS did not reveal significant genetic correlation results [22], this study revealed a significant genetic correlation between pain and educational attainment, consistent with the PRS association findings. We had expected to identify a causal relationship from SAs to SD. However, the absence of a significant causal relationship in the current study may reflect limitations in statistical power for the GWAS for both SA and SD summary statistics, as few genome-wide significant findings have been reported to date. However, the non-significance of this relationship may alternatively reflect the observation that the majority of people who die by suicide die on their first attempt [93, 94]. Thus, a large fraction of individuals who completed suicides would be expected to not have a prior attempt. Conversely, among patients who require medical attention for a suicide attempt only a relatively small fraction die on the first attempt [94].

The study has a few limitations. Even though this is one of the largest GWAS analyses for SD, the effective sample size is still more modest than previous SD GWAS or SA GWAS reported by ISGC. Even though we genotyped an additional ~1200 SD cases, the net increase in SD case sample size was >300 after case–control matching, while the control sample size was smaller compared to the previous SD GWAS. However, this SD GWAS is the first well-powered GWAS to leverage matching arrays across cases and controls, which we believe is an important strength. Secondly, we prioritized depression and SAs for conditional analysis. There are certainly other psychiatric conditions that warranted such a test and therefore the conditional test is not exhaustive. In addition, the summary statistics from Howard et al. are likely to be powerful enough for conditional analysis given that 102 independent variants were discovered, while the summary statistics from the ISGC analysis may not be powerful enough as the genome-wide significant loci are just beginning to be unveiled. Thirdly, even though we tried to provide replication evidence for suggestive association findings from ISGC, the samples used in this study partially overlapped with the samples in ISGC, and therefore are not completely independent. The Finngen replication of ISGC and MVP meta-analysis results on the other hand are completely independent. The GSMR analysis is just the beginning to explore the intricate relationship between these traits. The selection of SNPs used a relaxed *p* value threshold simply due to the limited power of available GWAS summary statistics and this certainly benefits from future growth in sample size.

## Supplementary information

Supplementary Text

Supplementary Table

## Data Availability

PLINK 1.07 https://zzz.bwh.harvard.edu/plink/ PLINK 1.9 https://www.cog-genomics.org/plink/ RICOPILI https://sites.google.com/a/broadinstitute.org/ricopili GEMMA https://github.com/genetics-statistics/GEMMA BOLT https://alkesgroup.broadinstitute.org/BOLT-LMM/BOLT-LMM_manual.html SAIGE https://github.com/weizhouUMICH/SAIGE PRSice-2 https://www.prsice.info/ LDSC https://github.com/bulik/ldsc GCTA https://yanglab.westlake.edu.cn/software/gcta/#Overview METAL https://genome.sph.umich.edu/wiki/METAL_Documentation FUMA https://fuma.ctglab.nl/ MAGMA https://ctg.cncr.nl/software/magma.
