# Supplementary material for: Genome-wide association study meta-analysis of suicide death and suicidal behavior
Source: Mol Psychiatry. 2022 Oct 17;28(2):891–900. doi: 10.1038/s41380-022-01828-9 (PMC9908547; doi:10.1038/s41380-022-01828-9)
Supplement: Supplementary file 1 — Supplementary Text [file 41380_2022_1828_MOESM1_ESM.docx]

**Genome-wide association study meta-analysis of suicide death and suicidal behavior**

Qingqin S Li, Ph.D.^1,2,*^, Andrey A. Shabalin, Ph.D.^3^, Emily DiBlasi, Ph.D.^3^, Srihari Gopal, M.D.^1^, Carla M Canuso, M.D.^1^, FinnGen, International Suicide Genetics Consortium, Aarno Palotie, M.D., Ph.D.^4^, Wayne C. Drevets, M.D.^4^, Anna R. Docherty, Ph.D.^3,5,6^, Hilary Coon, Ph.D.^3^

Members of International Suicide Genetics Consortium:

Niamh Mullins 1, 2, Jooeun Kang 3, Adrian I Campos 4, 5, Jonathan R I Coleman 6, 7, Alexis C Edwards 8, Hanga Galfalvy 9, 10, Daniel F Levey 11, 12, Adriana Lori 13, Andrey Shabalin 14, Anna Starnawska 15, 16, 17, 18, Mei-Hsin Su 19, Hunna J Watson 20, 21, 22, Mark Adams 23, Swapnil Awasthi 24, Michael Gandal 25, Jonathan D Hafferty 23, Akitoyo Hishimoto 26, Minsoo Kim 25, Satoshi Okazaki 27, Ikuo Otsuka 10, 27, Stephan Ripke 24, 28, 29, Erin B Ware 30, 31, Andrew W Bergen 32, 33, Wade H Berrettini 34, Martin Bohus 35, Harry Brandt 36, Xiao Chang 37, Wei J Chen 19, 38, 39, Hsi-Chung Chen 39, Steven Crawford 36, Scott Crow 40, Emily DiBlasi 14, Philibert Duriez 41, 42, Fernando Fernández-Aranda 43, Manfred M Fichter 44, 45, Steven Gallinger 46, Stephen J Glatt 47, Philip Gorwood 41, 42, Yiran Guo 37, Hakon Hakonarson 37, 48, Katherine A Halmi 49, Hai-Gwo Hwu 50, Sonia Jain 51, Stéphane Jamain 52, Susana Jiménez-Murcia 43, Craig Johnson 53, Allan S Kaplan 54, 55, 56, Walter H Kaye 57, Pamela K Keel 58, James L Kennedy 54, 55, 56, Kelly L Klump 59, Dong Li 37, Shih-Cheng Liao 39, Klaus Lieb 60, Lisa Lilenfeld 61, Chih-Min Liu 39, Pierre J Magistretti 62, 63, Christian R Marshall 64, James E Mitchell 65, Eric T Monson 14, Richard M Myers 66, Dalila Pinto 1, 2, Abigail Powers 13, Nicolas Ramoz 42, Stefan Roepke 67, Vsevolod Rozanov 68, 69, Stephen W Scherer 70, Christian Schmahl 35, Marcus Sokolowski 71, Michael Strober 72, 73, Laura M Thornton 22, Janet Treasure 74, 75, Ming T Tsuang 76, Stephanie H Witt 77, D Blake Woodside 55, 56, 78, 79, Zeynep Yilmaz 22, 80, 81, Lea Zillich 77, Rolf Adolfsson 82, Ingrid Agartz 83, 84, 85, Tracy M Air 86, Martin Alda 87, 88, Lars Alfredsson 89, 90, Ole A Andreassen 91, 92, Adebayo Anjorin 93, Vivek Appadurai 94, 95, María Soler Artigas 96, 97, 98, 99, Sandra Van der Auwera 100, M Helena Azevedo 101, Nicholas Bass 102, Claiton HD Bau 103, 104, Bernhard T Baune 105, 106, Frank Bellivier 107, 108, 109, 110, Klaus Berger 111, Joanna M Biernacka 112, Tim B Bigdeli 113, 114, Elisabeth B Binder 13, 115, Michael Boehnke 116, Marco P Boks 117, Rosa Bosch 96, 97, 118, David L Braff 119, Richard Bryant 120, Monika Budde 121, Enda M Byrne 122, 123, Wiepke Cahn 124, Miguel Casas 96, 97, 99, 118, Enrique Castelao 125, Jorge A Cervilla 126, Boris Chaumette 127, 128, 129, Sven Cichon 130, 131, 132, 133, Aiden Corvin 134, Nicholas Craddock 135, David Craig 136, Franziska Degenhardt 133, Srdjan Djurovic 137, 138, Howard J Edenberg 139, 140, Ayman H Fanous 113, 114, Jerome C Foo 141, Andreas J Forstner 130, 133, 142, Mark Frye 143, Janice M Fullerton 144, 145, Justine M Gatt 120, 144, Pablo V Gejman 146, 147, Ina Giegling 148, 149, Hans J Grabe 100, Melissa J Green 144, 150, Eugenio H Grevet 151, 152, Maria Grigoroiu-Serbanescu 153, Blanca Gutierrez 154, Jose Guzman-Parra 155, Steven P Hamilton 156, Marian L Hamshere 135, Annette Hartmann 148, Joanna Hauser 157, Stefanie Heilmann-Heimbach 133, Per Hoffmann 131, 132, 133, Marcus Ising 158, Ian Jones 135, Lisa A Jones 159, Lina Jonsson 160, René S Kahn 2, 161, John R Kelsoe 119, 162, Kenneth S Kendler 114, Stefan Kloiber 54, 158, 163, Karestan C Koenen 164, 165, 166, Manolis Kogevinas 167, Bettina Konte 148, Marie-Odile Krebs 127, 128, 129, Mikael Landén 160, 168, Jacob Lawrence 169, Marion Leboyer 107, 170, 171, Phil H Lee 28, 29, 172, Douglas F Levinson 173, Calwing Liao 174, 175, Jolanta Lissowska 176, Susanne Lucae 158, Fermin Mayoral 155, Susan L McElroy 177, Patrick McGrath 178, Peter McGuffin 7, Andrew McQuillin 102, Sarah Medland 179, Divya Mehta 180, 181, Ingrid Melle 91, 182, Yuri Milaneschi 183, Philip B Mitchell 150, Esther Molina 184, Gunnar Morken 185, 186, Preben Bo Mortensen 16, 80, 95, 187, Bertram Müller-Myhsok 115, 188, 189, Caroline Nievergelt 119, Vishwajit Nimgaonkar 190, Markus M Nöthen 133, Michael C O'Donovan 135, Roel A Ophoff 191, 192, Michael J Owen 135, Carlos Pato 193, 194, Michele T Pato 194, Brenda WJH Penninx 183, 183, Jonathan Pimm 102, Giorgio Pistis 125, James B Potash 195, Robert A Power 7, 196, 197, Martin Preisig 125, Digby Quested 198, Josep Antoni Ramos-Quiroga 96, 97, 99, 118, Andreas Reif 199, Marta Ribasés 96, 97, 98, 99, Vanesa Richarte 96, 97, 118, Marcella Rietschel 200, Margarita Rivera 7, 201, Andrea Roberts 202, Gloria Roberts 150, Guy A Rouleau 175, 203, Diego L Rovaris 204, Dan Rujescu 148, Cristina Sánchez-Mora 96, 97, 98, 99, Alan R Sanders 146, 147, Peter R Schofield 144, 145, Thomas G Schulze 121, 141, 205, 206, 207, Laura J Scott 116, Alessandro Serretti 208, Jianxin Shi 209, Stanley I Shyn 210, Lea Sirignano 141, Pamela Sklar 1, 2, 211, Olav B Smeland 91, 92, Jordan W Smoller 28, 166, 212, Edmund J S Sonuga-Barke 213, Gianfranco Spalletta 214, 215, John S Strauss 54, 163, Beata Świątkowska 216, Maciej Trzaskowski 122, Gustavo Turecki 217, Laura Vilar-Ribó 96, 99, John B Vincent 218, Henry Völzke 219, James TR Walters 135, Cynthia Shannon Weickert 144, 150, Thomas W Weickert 144, 150, Myrna M Weissman 220, 221, Leanne M Williams 222, Naomi R Wray 122, 181, Clement C Zai 28, 163, 165, 223, 224, 225, Allison E. Ashley-Koch 226, Jean C. Beckham 227, 228, Elizabeth R. Hauser 226, 229, Michael A. Hauser 226, Nathan A. Kimbrel 227, 228, Jennifer H. Lindquist 230, Benjamin McMahon 231, David W. Oslin 232, 233, Xuejun Qin 226, Esben Agerbo 80, 187, 234, Anders D Børglum 15, 16, 17, 18, Gerome Breen 6, 7, Annette Erlangsen 18, 235, 236, 237, Tõnu Esko 238, 239, Joel Gelernter 11, 12, David M Hougaard 234, 240, Ronald C Kessler 241, Henry R Kranzler 242, 243, Qingqin S Li 244, Nicholas G Martin 245, Andrew M McIntosh 23, Sarah E Medland 245, Ole Mors 234, 246, Merete Nordentoft 234, 247, Catherine M Olsen 248, David Porteous 249, Robert J Ursano 250, Danuta Wasserman 71, Thomas Werge 94, 234, 251, 252, David C Whiteman 248, Cynthia M Bulik 22, 168, 253, Hilary Coon 14, 254, Ditte Demontis 15, 16, 17, 18, Anna R Docherty 8, 14, Po-Hsiu Kuo 19, 39, Cathryn M Lewis 7, 255, J John Mann 256, Miguel E Rentería 4, 5, Daniel J Smith 257, Eli A Stahl 1, 2, 238, Murray B Stein 258, Fabian Streit 77, Virginia Willour 259, Douglas M Ruderfer 3, 260, 261

1, Department of Genetics and Genomic Sciences, Icahn School of Medicine at Mount Sinai, New York, NY, US

2, Department of Psychiatry, Icahn School of Medicine at Mount Sinai, New York, NY, US

3, Division of Genetic Medicine, Department of Medicine, Vanderbilt Genetics Institute, Vanderbilt University Medical Center, Nashville, TN, US

4, Department of Genetics and Computational Biology, QIMR Berghofer Medical Research Institute, Brisbane, QLD, Australia

5, School of Biomedical Sciences, Faculty of Medicine, The University of Queensland, Brisbane, QLD, Australia

6, National Institute for Health Research (NIHR) Maudsley Biomedical Research Centre at South London and Maudsley NHS Foundation Trust, King's College London, London, UK

7, Social Genetic and Developmental Psychiatry Centre, King's College London, London, UK

8, Department of Psychiatry, Virginia Commonwealth University, Richmond, VA, US

9, Department of Biostatistics, Columbia University, New York, NY, US

10, Department of Psychiatry, Columbia University, New York, NY, US

11, Department of Psychiatry, Veterans Affairs Connecticut Healthcare Center, West Haven, CT, US

12, Division of Human Genetics, Department of Psychiatry, Yale University School of Medicine, New Haven, CT, US

13, Department of Psychiatry and Behavioral Sciences, Emory University School of Medicine, Atlanta, GA, US

14, Department of Psychiatry, University of Utah School of Medicine, Salt Lake City, UT, US

15, Centre for Genomics and Personalized Medicine, CGPM, Aarhus University, Aarhus, Denmark

16, Centre for Integrative Sequencing, iSEQ, Aarhus University, Aarhus, Denmark

17, Department of Biomedicine, Aarhus University, Aarhus, Denmark

18, The Lundbeck Foundation Initiative for Integrative Psychiatric Research, iPSYCH, Aarhus University, Aarhus, Denmark

19, Institute of Epidemiology and Preventive Medicine, College of Public Health, National Taiwan University, Taipei, Taiwan

20, School of Psychology, Curtin University, Perth, Western Australia, Australia

21, Division of Paediatrics, The University of Western Australia, Perth, Western Australia, Australia

22, Department of Psychiatry, University of North Carolina at Chapel Hill, Chapel Hill, NC, US

23, Division of Psychiatry, University of Edinburgh, Edinburgh, UK

24, Department of Psychiatry and Psychotherapy, Charité - Universitätsmedizin Berlin, Berlin, Germany

25, Department of Psychiatry and Biobehavioral Science, Semel Institute, David Geffen School of Medicine, University of California, Los Angeles, Los Angeles, CA, US

26, Department of Psychiatry, Yokohama City University Graduate School of Medicine, Yokohama, Japan

27, Department of Psychiatry, Kobe University Graduate School of Medicine, Kobe, Japan

28, Stanley Center for Psychiatric Research, Broad Institute, Cambridge, MA, US

29, Analytical and Translational Genetics Unit, Massachusetts General Hospital, Boston, MA, US

30, Population Studies Center, Institute for Social Research, University of Michigan, Ann Arbor, MI, US

31, Survery Research Center, Institute for Social Research, University of Michigan, Ann Arbor, MI, US

32, BioRealm, LLC, Walnut, CA, US

33, Oregon Research Institute, Eugene, OR, US

34, Department of Psychiatry, Center for Neurobiology and Behavior, Perelman School of Medicine at the University of Pennsylvania, Philadelphia, PA, US

35, Department of Psychosomatic Medicine and Psychotherapy, Central Institute of Mental Health, Medical Faculty Mannheim, University of Heidelberg, Mannheim, Germany

36, The Center for Eating Disorders at Sheppard Pratt, Baltimore, MD, US

37, Center for Applied Genomics, Children's Hospital of Philadelphia, Philadelphia, PA, US

38, Center for Neuropsychiatric Research, National Health Research Institutes, Miaoli County, Taiwan

39, Department of Psychiatry, National Taiwan University Hospital, Taipei, Taiwan

40, Department of Psychiatry, University of Minnesota, Minneapolis, MN, US

41, Hôpital Sainte Anne, GHU Paris Psychiatrie et Neurosciences, Paris, France

42, Institute of Psychiatry and Neuroscience of Paris (IPNP), INSERM U1266, Université de Paris, Paris, France

43, Department of Psychiatry, University Hospital Bellvitge-IDIBELL and CIBEROBN, Barcelona, Spain

44, Department of Psychiatry and Psychotherapy, Ludwig-Maximilians-University (LMU), Munich, Germany

45, Schön Klinik Roseneck affiliated with the Medical Faculty of the University of Munich (LMU), Munich, Germany

46, Department of Surgery, Faculty of Medicine, University of Toronto, Toronto, Canada

47, Department of Psychiatry and Behavioral Sciences, SUNY Upstate Medical University, Syracuse, NY, US

48, The Perelman School of Medicine, University of Pennsylvania, Philadelphia, PA, US

49, Department of Psychiatry, Weill Cornell Medical College, New York, NY, US

50, Department of Psychiatry, National Taiwan University Hospital and College of Medicine, Taipei, Taiwan

51, Biostatistics Research Center, Herbert Wertheim School of Public Health and Human Longevity Science, University of California San Diego, La Jolla, CA, US

52, Inserm U955, Institut Mondor de recherches Biomédicales, Laboratoire, Neuro-Psychiatrie Translationnelle, and Fédération Hospitalo-Universitaire de Précision Médecine en Addictologie et Psychiatrie (FHU ADAPT), University Paris-Est-Créteil, Créteil, France

53, Eating Recovery Center, Denver, CO, US

54, Centre for Addiction and Mental Health, Toronto, ON, Canada

55, Department of Psychiatry, University of Toronto, Toronto, Canada

56, Institute of Medical Science, University of Toronto, Toronto, Canada

57, Department of Psychiatry, University of California San Diego, San Diego, CA, US

58, Department of Psychology, Florida State University, Tallahassee, FL, US

59, Department of Psychology, Michigan State University, Lansing, MI, US

60, Department of Psychiatry and Psychotherapy, University Medical Center, Mainz, Germany

61, Department of Clinical Psychology, The Chicago School of Professional Psychology, Washington DC, Washington, DC, US

62, BESE Division, King Abdullah University of Science and Technology, Thuwal, Saudi Arabia

63, Department of Psychiatry, University of Lausanne-University Hospital of Lausanne (UNIL-CHUV), Lausanne, Switzerland

64, Department of Paediatric Laboratory Medicine, The Hospital for Sick Children, Toronto, Canada

65, Department of Psychiatry and Behavioral Science, University of North Dakota School of Medicine and Health Sciences, Fargo, ND, US

66, HudsonAlpha Institute for Biotechnology, Huntsville, AL, US

67, Department of Psychiatry, Charité - Universitätsmedizin Berlin, Corporate Member of Freie Universität Berlin, Humboldt-Universität zu Berlin, Berlin Institute of Health, Campus Benjamin Franklin, Berlin, Germany

68, Department of Psychology, Saint-Petersburg State University, Saint-Petersburg, Russian Federation

69, Department of Borderline Disorders and Psychotherapy, V.M. Bekhterev National Medical Research Center for Psychiatry and Neurology, Saint-Petersburg, Russian Federation

70, Department of Genetics and Genomic Biology, The Hospital for Sick Children, Toronto, Canada

71, National Centre for Suicide Research and Prevention of Mental Ill-Health (NASP), LIME, Karolinska Institutet, Stockholm, Sweden

72, David Geffen School of Medicine, University of California Los Angeles, Los Angeles, LA, US

73, Department of Psychiatry and Biobehavioral Science, Semel Institute for Neuroscience and Human Behavior, University of California Los Angeles, Los Angeles, LA, US

74, Institute of Psychiatry, Psychology and Neuroscience, Department of Psychological Medicine, King’s College London, London, UK

75, National Institute for Health Research Biomedical Research Centre, King’s College London and South London and Maudsley National Health Service Foundation Trust, London, UK

76, Center for Behavioral Genomics, Department of Psychiatry, University of California, San Diego, San Diego, CA, US

77, Department of Genetic Epidemiology in Psychiatry, Central Institute of Mental Health, Medical Faculty Mannheim, University of Heidelberg, Mannheim, Germany

78, Centre for Mental Health, University Health Network, Toronto, Canada

79, Program for Eating Disorders, University Health Network, Toronto, Canada

80, National Centre for Register-Based Research, Aarhus University, Aarhus, Denmark

81, Department of Genetics, University of North Carolina at Chapel Hill, Chapel Hill, NC, US

82, Department of Clinical Sciences, Psychiatry, Umeå University Medical Faculty, Umeå, Sweden

83, Department of Psychiatric Research, Diakonhjemmet Hospital, Oslo, Norway

84, Department of Clinical Neuroscience, Centre for Psychiatry Research, Karolinska Institutet, Stockholm, Sweden

85, NORMENT, Institute of Clinical Medicine, University of Oslo, Oslo, Norway

86, Discipline of Psychiatry, University of Adelaide, Adelaide, SA, Australia

87, Department of Psychiatry, Dalhousie University, Halifax, NS, Canada

88, National Institute of Mental Health, Klecany, CZ

89, Department of Clinical Neuroscience, Karolinska Institutet, Stockholm, Sweden

90, Inst of Environmental Medicine, Karolinska Institutet, Stockholm, Sweden

91, Division of Mental Health and Addiction, Oslo University Hospital, Oslo, Norway

92, NORMENT, University of Oslo, Oslo, Norway

93, Psychiatry, Berkshire Healthcare NHS Foundation Trust, Bracknell, UK

94, Institute of Biological Psychiatry, Copenhagen Mental Health Services, Copenhagen University Hospital, Copenhagen, Denmark

95, The Lundbeck Foundation Initiative for Integrative Psychiatric Research, iPSYCH, Copenhagen, Denmark

96, Department of Psychiatry, Hospital Universitari Vall d’Hebron, Barcelona, Spain

97, Biomedical Network Research Centre on Mental Health (CIBERSAM), Instituto de Salud Carlos III, Madrid, Spain

98, Department of Genetics, Microbiology & Statistics, University of Barcelona, Barcelona, Spain

99, Psychiatric Genetics Unit, Group of Psychiatry, Mental Health and Addiction, Vall d’Hebron Research Institute (VHIR), Universitat Autònoma de Barcelona, Barcelona, Spain

100, Department of Psychiatry and Psychotherapy, University Medicine Greifswald, Greifswald, Mecklenburg-Vorpommern, Germany

101, Department of Psychiatry, University of Coimbra, Coimbra, Portugal

102, Division of Psychiatry, University College London, London, UK

103, Laboratory of Developmental Psychiatry, Hospital de Clínicas de Porto Alegre, Porto Alegre, RS, Brazil

104, Department of Genetics, Universidade Federal do Rio Grande do Sul, Porto Alegre, RS, Brazil

105, Department of Psychiatry, Melbourne Medical School, University of Melbourne, Melbourne, Australia

106, Department of Psychiatry, University of Münster, Münster, Germany

107, Department of Psychiatry and Addiction Medicine, Assistance Publique - Hôpitaux de Paris, Paris, France

108, Paris Bipolar and TRD Expert Centres, FondaMental Foundation, Paris, France

109, UMR-S1144 Team 1 : Biomarkers of relapse and therapeutic response in addiction and mood disorders, INSERM, Paris, France

110, Psychiatry, Université Paris Diderot, Paris, France

111, Institute of Epidemiology and Social Medicine, University of Münster, Münster, Nordrhein-Westfalen, Germany

112, Health Sciences Research, Mayo Clinic, Rochester, MN, US

113, Department of Psychiatry and Behavioral Sciences, State University of New York Downstate Medical Center, New York, NY, US

114, Department of Psychiatry, Virginia Commonwealth University, Richmond, VA, US

115, Department of Translational Research in Psychiatry, Max Planck Institute of Psychiatry, Munich, Germany

116, Center for Statistical Genetics and Department of Biostatistics, University of Michigan, Ann Arbor, MI, US

117, Psychiatry, UMC Utrecht Hersencentrum, Utrecht, Netherlands

118, Department of Psychiatry and Legal Medicine, Universitat Autònoma de Barcelona, Barcelona, Spain

119, Department of Psychiatry, University of California San Diego, La Jolla, CA, US

120, School of Psychology, University of New South Wales, Sydney, NSW, Australia

121, Institute of Psychiatric Phenomics and Genomics (IPPG), University Hospital, LMU Munich, Munich, Germany

122, Institute for Molecular Bioscience, The University of Queensland, Brisbane, QLD, Australia

123, Centre for Children’s Health Research, The University of Queensland, Brisbane, QLD, Australia

124, Department of Psychiatry, UMC Utrecht Hersencentrum Rudolf Magnus, Utrecht, Netherlands

125, Department of Psychiatry, Lausanne University Hospital and University of Lausanne, Lausanne, Vaud, Switzerland

126, Mental Health Unit, Department of Psychiatry, Faculty of Medicine, Granada University Hospital Complex, University of Granada, Granada, Spain

127, Institut de Psychiatrie, CNRS GDR 3557, Paris, France

128, Department of Evaluation, Prevention and Therapeutic innovation, GHU Paris Psychiatrie et Neurosciences, Paris, France

129, Team Pathophysiology of psychiatric diseases, Université de Paris, Institute of Psychiatry and Neuroscience of Paris (IPNP), INSERM U1266, Paris, France

130, Institute of Neuroscience and Medicine (INM-1), Research Centre Jülich, Jülich, Germany

131, Institute of Medical Genetics and Pathology, University Hospital Basel, Basel, Switzerland

132, Department of Biomedicine, University of Basel, Basel, Switzerland

133, Institute of Human Genetics, University of Bonn, School of Medicine & University Hospital Bonn, Bonn, Germany

134, Neuropsychiatric Genetics Research Group, Dept of Psychiatry and Trinity Translational Medicine Institute, Trinity College Dublin, Dublin, Ireland

135, Medical Research Council Centre for Neuropsychiatric Genetics and Genomics, Division of Psychological Medicine and Clinical Neurosciences, Cardiff University, Cardiff, UK

136, Department of Translational Genomics, University of Southern California, Pasadena, CA, US

137, Department of Medical Genetics, Oslo University Hospital, Oslo, Norway

138, NORMENT, KG Jebsen Centre for Psychosis Research, Department of Clinical Science, University of Bergen, Bergen, Norway

139, Department of Medical & Molecular Genetics, Indiana University, Indianapolis, IN, US

140, Biochemistry and Molecular Biology, Indiana University School of Medicine, Indianapolis, IN, US

141, Department of Genetic Epidemiology in Psychiatry, Central Institute of Mental Health, Medical Faculty Mannheim, Heidelberg University, Mannheim, Germany

142, Centre for Human Genetics, University of Marburg, Marburg, Germany

143, Department of Psychiatry & Psychology, Mayo Clinic, Rochester, MN, US

144, Neuroscience Research Australia, Sydney, NSW, Australia

145, School of Medical Sciences, University of New South Wales, Sydney, NSW, Australia

146, Department of Psychiatry and Behavioral Sciences, NorthShore University HealthSystem, Evanston, IL, US

147, Department of Psychiatry and Behavioral Neuroscience, University of Chicago, Chicago, IL, US

148, Dept. of Psychiatry, Psychotherapy and Psychosomatics, Martin-Luther-University Halle-Wittenberg, Halle (Saale), Germany

149, Department of Psychiatry, University of Munich, Munich, Germany

150, School of Psychiatry, University of New South Wales, Sydney, NSW, Australia

151, ADHD Outpatient Program, Adult Division, Hospital de Clínicas de Porto Alegre, Porto Alegre, RS, Brazil

152, Department of Psychiatry, Universidade Federal do Rio Grande do Sul, Porto Alegre, RS, Brazil

153, Biometric Psychiatric Genetics Research Unit, Alexandru Obregia Clinical Psychiatric Hospital, Bucharest, Romania

154, Department of Psychiatry, Faculty of Medicine and Biomedical Research Centre (CIBM), University of Granada, Granada, Spain

155, Mental Health Department, University Regional Hospital. Biomedicine Institute (IBIMA), Málaga, Spain

156, Psychiatry, Kaiser Permanente Northern California, San Francisco, CA, US

157, Department of Psychiatry, Laboratory of Psychiatric Genetics, Poznan University of Medical Sciences, Poznan, Poland

158, Max Planck Institute of Psychiatry, Munich, Germany

159, Department of Psychological Medicine, University of Worcester, Worcester, UK

160, Department of Psychiatry and Neuroscience, University of Gothenburg, Gothenburg, Sweden

161, Psychiatry, UMC Utrecht Hersencentrum Rudolf Magnus, Utrecht, Netherlands

162, Institute for Genomic Medicine, University of California San Diego, La Jolla, CA, US

163, Department of Psychiatry, University of Toronto, Toronto, ON, Canada

164, Stanley Center for Psychiatric Research, Broad Institute, Cambridge, MA, US

165, Department of Epidemiology, Harvard TH Chan School of Public Health, Boston, MA, US

166, Department of Psychiatry, Massachusetts General Hospital, Boston, MA, US

167, Center for Research in Environmental Epidemiology (CREAL), Barcelona, Spain

168, Department of Medical Epidemiology and Biostatistics, Karolinska Institutet, Stockholm, Sweden

169, Psychiatry, North East London NHS Foundation Trust, Ilford, UK

170, INSERM, Paris, France

171, Faculté de Médecine, Université Paris Est, Créteil, France

172, Psychiatric and Neurodevelopmental Genetics Unit, Massachusetts General Hospital, Boston, MA, US

173, Psychiatry & Behavioral Sciences, Stanford University, Stanford, CA, US

174, Department of Human Genetics, McGill University, Montreal, QC, Canada

175, Montreal Neurological Institute and Hospital, Montreal, QC, Canada

176, Cancer Epidemiology and Prevention, M. Sklodowska-Curie Cancer Center and Institute of Oncology, Warsaw, Poland

177, Research Institute, Lindner Center of HOPE, Mason, OH, US

178, Psychiatry, Columbia University College of Physicians and Surgeons, New York, NY, US

179, Genetics and Computational Biology, QIMR Berghofer Medical Research Institute, Brisbane, QLD, Australia

180, School of Psychology and Counseling, Queensland University of Technology, Brisbane, QLD, Australia

181, Queensland Brain Institute, The University of Queensland, Brisbane, QLD, Australia

182, Division of Mental Health and Addiction, University of Oslo, Institute of Clinical Medicine, Oslo, Norway

183, Department of Psychiatry, Amsterdam UMC, Vrije Universiteit and GGZ inGeest, Amsterdam, Netherlands

184, Department of Nursing, Faculty of Health Sciences and Biomedical Research Centre (CIBM), University of Granada, Granada, Spain

185, Mental Health, Faculty of Medicine and Health Sciences, Norwegian University of Science and Technology - NTNU, Trondheim, Norway

186, Psychiatry, St Olavs University Hospital, Trondheim, Norway

187, Centre for Integrated Register-based Research, Aarhus University, Aarhus, Denmark

188, Munich Cluster for Systems Neurology (SyNergy), Munich, Germany

189, University of Liverpool, Liverpool, UK

190, Psychiatry and Human Genetics, University of Pittsburgh, Pittsburgh, PA, US

191, Psychiatry, Erasmus University Medical Center, Rotterdam, Netherlands

192, Jane and Terry Semel Institute for Neuroscience and Human Behavior, Los Angeles, CA, US

193, College of Medicine Institute for Genomic Health, SUNY Downstate Medical Center College of Medicine, Brooklyn, NY, US

194, Institute for Genomic Health, SUNY Downstate Medical Center College of Medicine, Brooklyn, NY, US

195, Psychiatry, University of Iowa, Iowa City, IA, US

196, Genetics, BioMarin Pharmaceuticals, London, UK

197, St Edmund Hall, University of Oxford, Oxford, UK

198, Department of Psychiatry, University of Oxford, Oxford, UK

199, Department of Psychiatry, Psychosomatic Medicine and Psychotherapy, University Hospital Frankfurt, Frankfurt, Germany

200, Department of Genetic Epidemiology in Psychiatry, Central Institute of Mental Health, Medical Faculty Mannheim, Heidelberg University, Mannheim, Baden-Württemberg, Germany

201, Department of Biochemistry and Molecular Biology II and Institute of Neurosciences, Biomedical Research Centre (CIBM), University of Granada, Granada, Spain

202, Department of Environmental Health, Harvard TH Chan School of Public Health, Boston, MA, US

203, Department of Neurology and Neurosurgery, McGill University, Faculty of Medicine, Montreal, QC, Canada

204, Department of Physiology and Biophysics, Instituto de Ciencias Biomedicas Universidade de Sao Paulo, São Paulo, SP, Brazil

205, Department of Psychiatry and Behavioral Sciences, Johns Hopkins University School of Medicine, Baltimore, MD, US

206, Human Genetics Branch, Intramural Research Program, National Institute of Mental Health, Bethesda, MD, US

207, Department of Psychiatry and Psychotherapy, University Medical Center Göttingen, Göttingen, Germany

208, Department of Biomedical and NeuroMotor Sciences, University of Bologna, Bologna, Italy

209, Division of Cancer Epidemiology and Genetics, National Cancer Institute, Bethesda, MD, US

210, Behavioral Health Services, Kaiser Permanente Washington, Seattle, WA, US

211, Department of Neuroscience, Icahn School of Medicine at Mount Sinai, New York, NY, US

212, Psychiatric and Neurodevelopmental Genetics Unit (PNGU), Massachusetts General Hospital, Boston, MA, US

213, Institute of Psychology, Psychiatry & Neuroscience, King's College London, London, UK

214, Menninger Department of Psychiatry and Behavioral Sciences, Baylor College of Medicine, Houston, Houston, TX, US

215, Laboratory of Neuropsychiatry, IRCCS Santa Lucia Foundation, Rome, Rome, Italy

216, Department of Environmental Epidemiology, Nofer Institute of Occupational Medicine, Lodz, Poland

217, Department of Psychiatry, McGill University, Montreal, QC, Canada

218, Molecular Brain Science, Centre for Addiction and Mental Health, Toronto, ON, Canada

219, Institute for Community Medicine, University Medicine Greifswald, Greifswald, Mecklenburg-Vorpommern, Germany

220, Columbia University College of Physicians and Surgeons, New York, NY, US

221, Division of Translational Epidemiology, New York State Psychiatric Institute, New York, NY, US

222, Department of Psychiatry and Behavioral Sciences, Stanford University, Stanford, CA, US

223, Institute of Medical Science, University of Toronto, Toronto, ON, Canada

224, Molecular Brain Science, Campbell Family Mental Health Research Institute, Centre for Addiction and Mental Health, Toronto, ON, Canada

225, Laboratory Medicine and Pathobiology, University of Toronto, Toronto, ON, Canada

226, Duke Molecular Physiology Institute, Duke University Medical Center, Durham, NC, USA

227, VISN 6 Mid-Atlantic Mental Illness Research, Education, and Clinical Center, Durham Veterans Affairs Health Care System, Durham, NC, USA

228, Department of Psychiatry and Behavioral Sciences, Duke University School of Medicine, Durham, NC, USA

229, Cooperative Studies Program Epidemiology Center, Durham Veterans Affairs Health Care System, Durham, NC, USA

230, VA Health Services Research and Development Center of Innovation to Accelerate Discovery and Practice Transformation, Durham Veterans Affairs Health Care System, Durham, NC, USA

231, Theoretical Division, Los Alamos National Laboratory, Los Alamos National Laboratory, Los Alamos, NM, USA

232, VISN 4 Mental Illness Research, Education, and Clinical Center, Corporal Michael J. Crescenz VA Medical Center, Philadelphia, PA, USA

233, Department of Psychiatry, Perelman School of Medicine, University of Pennsylvania, Philadelphia, PA, USA

234, The Lundbeck Foundation Initiative for Integrative Psychiatric Research, iPSYCH, Aarhus, Denmark

235, Center of Mental Health Research, Australian National University, Canberra, Australia

236, Department of Mental Health, Johns Hopkins Bloomberg School of Public Health, Baltimore, MD, US

237, Danish Research Institute for Suicide Prevention, Mental Health Centre Copenhagen, Copenhagen, Denmark

238, Program in Medical and Population Genetics, Broad Institute, Cambridge, MA, US

239, Estonian Genome Center, Institute of Genomics, University of Tartu, Tartu, Estonia

240, Center for Neonatal Screening, Department for Congenital Disorders, Statens Serum Institut, Copenhagen, Denmark

241, Department of Health Care Policy, Harvard Medical School, Boston, MA, US

242, Department of Psychiatry, University of Pennsylvania Perelman School of Medicine, Philadelphia, PA, US

243, VISN 4 MIRECC, Crescenz VAMC, Philadelphia, PA, US

244, Neuroscience, Janssen Research & Development, LLC, Titusville, NJ, US

245, Department of Genetics and Computational Biology, QIMR Berghofer Medical Research Institute, Herston, QLD, Australia

246, Psychosis Research Unit, Aarhus University Hospital, Risskov, Aarhus, Denmark

247, Mental Health Center Copenhagen, Copenhagen University Hospital, Copenhagen, Denmark

248, Department of Population Health, QIMR Berghofer Medical Research Institute, Herston, QLD, Australia

249, Institute for Genetics and Molecular Medicine, University of Edinburgh, Edinburgh, UK

250, Department of Psychiatry, Uniformed University of the Health Sciences, Bethesda, MD, US

251, Department of Clinical Medicine, University of Copenhagen, Copenhagen, Denmark

252, Lundbeck Foundation GeoGenetics Centre, GLOBE Institute, University of Copenhagen, Copenhagen, Denmark

253, Department of Nutrition, University of North Carolina at Chapel Hill, Chapel Hill, NC, US

254, Biomedical Informatics, University of Utah School of Medicine, Salt Lake City, UT, US

255, Department of Medical & Molecular Genetics, King's College London, London, UK

256, Departments of Psychiatry and Radiology, Columbia University, New York, NY, US

257, Institute of Health and Wellbeing, University of Glasgow, Glasgow, UK

258, Department of Psychiatry and School of Public Health, University of California San Diego, La Jolla, CA, US

259, Department of Psychiatry, University of Iowa, Iowa City, IA, US

260, Department of Biomedical Informatics, Vanderbilt University Medical Center, Nashville, TN, US

261, Department of Psychiatry and Behavioral Sciences, Vanderbilt University Medical Center, Nashville, TN, US

**Supplementary Method** **S1.** Additional details of cohorts and sample ascertainment

**Supplementary Method** **S2.** Additional genotyping details of the study cohorts

**Supplementary Method S3**. Additional details on QC, principal component analysis (PCA), case-control matching, and imputation

**Supplementary Method** **S4.** Additional details on the ISGC summary statistics used in the mtCOJO, genetic correlation and GSMR analysis

**Supplementary Method** **S5.** Cross-reference of this study results with other published GWAS results

**Supplementary Method** **S6.** Additional details on the summary statistics used in the PRS association analysis

**Supplementary Method** **S7.** Additional details on the summary statistics used in the genetic correlation analysis

**Supplementary Text S1**. Replication results.

**Supplementary Text S2.** Genetic correlation results for suicidal attempts using ISGC summary statistics (excluding death cohorts).

**Supplementary Text S3**. Discussion on suggestive gene-based associations.

**Supplementary Figure 1.** Step by step QC for SD cohorts. An iterative process of case-control matching in cohort 1 A-C and cohort 2 D-H and the resulting final samples in SD GWAS meta-analysis [Cohort 1 & 2 (I)].

**Supplementary Figure 2** QQ-plots for suicide death GWAS meta-analysis: **SNP-level associations** - (A), suicide death condition on MDD (suicide death | MDD, B); and suicide death condition on suicidal attempt (suicide death | attempt, C) and **gene-level associations** - suicide death (D), suicide death | MDD (E); and suicide death | attempt (F); and suicidal behavior GWAS meta-analysis. **SNP-level associations** for suicidal behavior (G) and suicidal behavior condition on MDD (suicidal behavior | MDD, H), and **gene-level associations** for suicidal behavior (I) and suicidal behavior | MDD (J).

**Supplementary Figure 3** Genome-wide significant association signals. Manhattan plots for SNP-level associations for suicide death | MDD (A); and suicide death | attempt (B), and gene-level associations for suicide death | MDD (C); and suicide death | attempt (D); and for suicidal behavior | MDD: SNP-level associations (E) and gene-level associations (F).

**Supplementary Figure 4** LocusZoom regional plots for additional loci for suicide death.

**Supplementary Figure 5** LocusZoom regional plots for additional loci for suicidal behavior.

**Supplementary Figure 6** GSMR analysis to test the bi-directional causal relationship between depression, suicidal attempt, and suicide death.

**Supplementary Figure 7** Expression level of SOX5 [source: https://www.ncbi.nlm.nih.gov/gene/6660]

**Supplementary Method** **S1.** Additional details of cohorts and sample ascertainment

A total of 5 cohorts were used in this study.

| **Cohort** | **Cases** | **Controls** |
| --- | --- | --- |
| 1 | Utah Suicide **death** wave 1-5 (n = 869) | Coriell controls screened for psychiatric medical and family history (n = 804) |
| 2 | Utah Suicide **death** Wave 6 + 7 (n = 2,896) | IAMDGC controls not screened for suicide attempt (n = 5,768) |
| 3 | Janssen suicidal **attempt** cases (n = 269) | Psychiatric controls within the same studies (n = 809) |
| 4 | Janssen suicidal **attempt** cases (n = 183) | Psychiatric controls within the same studies (n = 1,199) |
| 5 | FinnGen suicidal **attempt** cases (n ~ 4,098) | FinnGen controls (n = 247,898) |
| Total | **8,315** | **256,478** |

***Suicide death cases and controls***

A two-decade collaboration with the Utah Office of Medical Examiner (OME) has allowed for the ongoing state-wide collection of DNA samples from persons who died by suicide. The centralized OME and conservative determination maximized the accuracy of suicide case status ^1^. Sample collection, secure linkage to electronic health data, and subsequent study of genotype and de-identified phenotype data have been approved by the Institutional Review Boards of the University of Utah, the Utah Department of Health, and Intermountain Healthcare. Suicide cause-of-death determination results from a detailed OME investigation of the scene of the death and circumstances of death, determination of medical conditions by full autopsy, review of medical and other public records concerning the case, interviews with survivors, and standard toxicology workups. Suicide determination is made by the OME conservatively due to its impact on surviving relatives. DNA from suicide deaths was extracted from whole blood using the Qiagen Autopure LS automated DNA extractor (QIAGEN, Germantown, MD). Genotyping is described below.

To date, 6,080 samples have been attempted for genotyping in three waves (See Supplementary Method S2 for detailed information on sample collection). Wave 1 and 2 samples were included in a previous report ^2^ except that the cases were matched to different sets of controls (Generation Scotland samples genotyped using OmniExpress and UK10K samples with whole-genome sequencing data). After quality control procedures, de-duplication, and ancestry matching, data were divided into two cohorts based on the genotype facility/wave. Cohort 1 (Table 1) consisted of 869 samples genotyped in wave 1 matched to 804 control samples mostly from the Coriell Institute for Medical Research (Camden, NJ) and a small subset from BioIVT (Westbury, NY), both genotyped using a matching array platform. Coriell healthy control samples were drawn from NINDS Human Genetics Repository managed by the Coriell Institute for Medical Research. Control samples from BioIVT were self-reported to be free of psychiatric conditions. Cohort 2 (Table 1) consisted of 2,896 samples genotyped in wave 2/3 and matched to 5,768 control subjects genotyped using Infinium CoreExome array (Illumina, Inc., San Diego, CA) obtained from dbGaP study phs001039: International Age-Related Macular Degeneration Genomics Consortium (IAMDGC) AMD Exome Chip experiment ^3^. CoreExome array and PsychArray shared the identical CoreExome array backbone except that PsychArray carries additional ~50K SNPs selected by Psychiatric Genomic Consortium (PGC). The detailed step-by-step QC leading to the sample size described herein is provided in Supplementary Figure S1.

***Janssen suicidal attempt cases and psychiatric controls***

The Janssen lifetime suicide attempt cohort consists of subjects of European ancestry and was drawn from 12 clinical trial samples (NCT00044681, NCT00397033, NCT00412373, NCT00334126, NCT01193153, NCT02497287, NCT02422186, NCT01627782, NCT00253162, NCT00257075, NCT01515423, and NCT01529515) conducted by Janssen Research & Development, LLC. The lifetime suicide attempt history was based on detailed clinical interviews and medical records, or/and Columbia-Suicide Severity Rating Scale (C-SSRS). The psychiatric controls are patients enrolled in the same studies without endorsing lifetime suicidal attempts. A subset of samples was included in the ISGC meta-analysis^4^ and detailed in Supplementary Method S2). The samples were divided into two cohorts based on the genotyping platform. The first suicidal attempt cohort consisted of 269 suicidal attempters and 809 psychiatric controls from the same studies, while the second suicidal attempt cohort consisted of 183 suicidal attempters and 1199 psychiatric controls.

***The FinnGen Study***

The FinnGen Study is a Finnish, nationwide GWAS meta-analysis of 9 biobanks. The cohorts have been linked with longitudinal electronic health record (EHR) data from nationwide health registries. For the suicidal attempt analysis using FinnGen data release 6 (R6) from the FinnGen Study included 4,098 individuals with suicidal attempt history, defined as the presence of suicidal attempt International Classification of Diseases (ICD) codes and 247,898 individuals without the relevant codes. The diagnosis codes used to define suicidal attempt have been provided in Supplementary Table S1. Only the stringent codes listed were used to define suicidal attempt cases. Unlike [ICD-10-CM codes](https://www.icd10data.com/ICD10CM/Codes) such as T36.0X2 “Poisoning by penicillins, intentional self-harm” used to differentiate from T36.0X1 “Poisoning by penicillins, accidental (unintentional)” or “T36.0X4 Poisoning by penicillins, undetermined”, the corresponding FinnGen ICD-10 codes mostly had 2 digit resolution, such as T36. Likewise, ICD-10-CM code T14.91 is used for suicide attempt, while T14.90 is for “injury, unspecified”. In FinnGen T14.9 is infrequently used (only 74 occurrence in the R6 release of detailed longitudinal data (v2.0). To avoid including true suicidal attempt cases in the control cohorts, the controls in the genetic analysis included only study participants without either stringent or loose suicidal attempt ICD codes (Supplementary Table S1).

Patients and control subjects in FinnGen provided informed consent for biobank research, based on the Finnish Biobank Act. Alternatively, separate research cohorts, collected prior the Finnish Biobank Act came into effect (in September 2013) and start of FinnGen (August 2017), were collected based on study-specific consents and later transferred to the Finnish biobanks after approval by Fimea, the National Supervisory Authority for Welfare and Health. Recruitment protocols followed the biobank protocols approved by Fimea. The Coordinating Ethics Committee of the Hospital District of Helsinki and Uusimaa (HUS) approved the FinnGen study protocol Number HUS/990/2017.

The FinnGen study is approved by Finnish Institute for Health and Welfare (permit numbers: THL/2031/6.02.00/2017, THL/1101/5.05.00/2017, THL/341/6.02.00/2018, THL/2222/6.02.00/2018, THL/283/6.02.00/2019, THL/1721/5.05.00/2019, THL/1524/5.05.00/2020, and THL/2364/14.02/2020), Digital and population data service agency (permit numbers: VRK43431/2017-3, VRK/6909/2018-3, VRK/4415/2019-3), the Social Insurance Institution (permit numbers: KELA 58/522/2017, KELA 131/522/2018, KELA 70/522/2019, KELA 98/522/2019, KELA 138/522/2019, KELA 2/522/2020, KELA 16/522/2020 and Statistics Finland (permit numbers: TK-53-1041-17 and TK-53-90-20).

The Biobank Access Decisions for FinnGen samples and data utilized in FinnGen Data Freeze 6 include: THL Biobank BB2017_55, BB2017_111, BB2018_19, BB_2018_34, BB_2018_67, BB2018_71, BB2019_7, BB2019_8, BB2019_26, BB2020_1, Finnish Red Cross Blood Service Biobank 7.12.2017, Helsinki Biobank HUS/359/2017, Auria Biobank AB17-5154, Biobank Borealis of Northern Finland_2017_1013, Biobank of Eastern Finland 1186/2018, Finnish Clinical Biobank Tampere MH0004, Central Finland Biobank 1-2017, and Terveystalo Biobank STB 2018001.

**Supplementary Method** **S2.** Additional genotyping details of the study cohorts

**Additional Details of Utah Sample Collection & Genotyping.** In the first wave we genotyped a total of 1,440 cases (288 samples each batch in a total of five batches), including samples from the beginning of the collection through January 2014. Funding limitations precluded genotyping of all samples. This wave 1 genotyping prioritized cases with the most complete available data from the Utah Population Database, including completeness of diagnostic and genealogical data. Subsequent collection and funding resources made wave 2 possible, and this genotyping included a total of 3196 samples not previously genotyped in wave 1, along with the additional, newer cases made available 2014-2017. Wave 3 samples added an additional 1444 samples.

**Details of Utah cases**

| Wave | Batch | Sample Size | PsychArray Version | Genotyping facility |
| --- | --- | --- | --- | --- |
| 1 | 1 | 288 | PsychArray-B | U of Utah genomic core |
| 1 | 2 | 288 | PsychArray-B | U of Utah genomic core |
| 1 | 3 | 288 | PsychArray-B | U of Utah genomic core |
| 1 | 4 | 288 | InfiniumPsychArray-24v1-1_A1 | U of Utah genomic core |
| 1 | 5 | 288 | PsychArray-B | U of Utah genomic core |
| 2 | 6 | 3196 | InfiniumPsychArray-24v1-2_A1 | Illumina, Inc. |
| 3 | 7 | 1444 |  | Illumina, Inc. |

**Additional Details of Janssen Sample Collection & Genotyping.** The Janssen lifetime suicide attempt cohort consists of subjects of European ancestry and was drawn from 12 clinical trial samples (NCT00044681, NCT00397033, NCT00412373, NCT00334126, NCT01193153, NCT02497287, NCT02422186, NCT01627782, NCT00253162, NCT00257075, NCT01515423, and NCT01529515) conducted by Janssen Research & Development, LLC. Detailed descriptions of these clinical trials can be found at ClinicalTrials.gov, as well as in published works ^5-22^. A subset of clinical trial samples (NCT00334126, NCT00397033, NCT00412373, and NCT00044681, NCT02497287, NCT02422186, NCT00253162, NCT00257075) was described previously ^23-31^. The clinical diagnoses of MDD, schizophrenia, schizoaffective, and bipolar disorder in Janssen clinical studies were based on expert clinician interviews conducted using DSM-IV-TR or DSM-V criteria. In two studies (NCT00397033 and NCT00412373), the diagnosis of schizoaffective disorder was confirmed using an interview-based SCID (Structured Clinical Interview for DSM-IV-TR). All samples were genotyped at Illumina, Inc. San Diego, CA facility.

| Internal identifier | Clinical trial ID | PMID | In Mullins et al. 2022 | Array platform | Cases | Controls | C-SSRS | Disease | Janssen Genotyping batch |
| --- | --- | --- | --- | --- | --- | --- | --- | --- | --- |
| **Janssen cohort #1** | | | | | | | | | |
| RIS-INT-93 | NCT00044681 | 16760927 | Yes | HumanOmni5Exome-4v1 | 85 | 318 |  | MDD | 3 |
| KETIV-TRD-2002 | NCT01627782 | 28086004 | No | HumanOmni5Exome-4v1 | 8 | 37 | Yes | MDD | 3 |
| R076477-SCA-3001 | NCT00397033 | 20492853, 20957127, 26934062 | Yes | Human1M-*DuoV3* | 24 | 66 |  | schizoaffective | 2 |
| R076477-SCA-3002 | NCT00412373 | 20814330, 20957127,  26934062 | Yes | Human1M-*DuoV3* | 40 | 49 |  | schizoaffective | 2 |
| RIS-INT-69 | NCT00253162 | 15572276 | No | Human1M-*DuoV3* | 22 | 168 |  | Bipolar | 2 |
| RIS-USA-239 | NCT00257075 | 15169694 | No | Human1M-*DuoV3* | 69 | 55 |  | Bipolar | 2 |
| R076477-SCH-3015 | NCT00334126 | 19411369 | Yes | Human1M-*DuoV3* | 20 | 87 |  | schizophrenia | 1 |
| **Janssen cohort #2** | | | | | | | | | |
| R092670-PSY-3011 | NCT01515423 | 32184607, 30994855 | No | PsychArray | 35 | 435 | Yes | schizophrenia | 9 |
| R092670-PSY-3012 | NCT01529515 | 25820612, 30994855 | No | PsychArray | 18 | 145 | Yes | schizophrenia | 9 |
| ESKETIN-TRD-3004 | NCT02497287 | 32316080 | No | PsychArray | 73 | 412 | Yes | MDD | 8a |
| ESKETIN-TRD-3005 | NCT02422186 | 31734084 | No | PsychArray | 14 | 80 | Yes | MDD | 8a |
| R092670-SCA-3004^a^ | NCT01193153 | 25562685, 28454922, 28005578,  26934062 | Yes | PsychArray | 44 | 129 | Yes | schizoaffective | 9 |
| **Controls for Utah Suicide death cohort #1** | | | | | | | | | |
| Coriell controls^b^ |  |  |  | PsychArray |  | 805 |  | Mostly healthy control some with medical history for mental health | 6 |

*The analysis in Mullins et al., 2021 did not use CSSR-S data.

^b^Coriell healthy control samples were drawn from NINDS Human Genetics Repository (neurologically normal Caucasian control panel NDPT020, NDPT079, NDPT084, NDPT090, NDPT093, NDPT094, NDPT095, NDPT096, NDPT098, and NDPT099) managed by the Coriell Institute for Medical Research.

**Supplementary Method S3**. Additional details on QC, principal component analysis (PCA), case-control matching, and imputation

***QC***

The initial QC was performed by genotyping batch before merging using PLINK^32^. SNPs with >5% missingness, minor allele frequency less than 1%, deviating from Hardy-Weinberg Equilibrium (*p* <0.000001) were removed. Samples with a call rate < 95% were also removed using a local QC pipeline. The QC process was repeated after merging using PLINK and further QC’ed using RICOPILI pipeline^33^. The detailed step-by-step QC using RICOPILI is provided in Supplementary Figure S1. Post-association genotyping quality control was performed using SNP clustering visualization via Genome Studio v2.0 (Illumina Inc., San Diego, CA <https://www.illumina.com/techniques/microarrays/array-data-analysisexperimentaldesign/genomestudio.html>) except FinnGen cohort and IAMDGC control samples where raw genotyping data are not available.

***Principal Component Analysis (PCA), Case-Control Matching, and Imputation***

The ancestry PCA was performed using EIGENSTRAT^34, 35^ as part of the RICOPILI pipeline. Case and control matching were performed iteratively within RICOPILI. The details of PCA matching using RICOPILI were documented in Supplementary Figure S1. Genotypes were subsequently imputed in matched cases and controls based on the SNPs common between cases and controls and using 1000 Genomes reference panel (ALL_v5b.20130502_1KG_0517) using minimac3^36^ and Eagle^37^ as implemented in the RICOPILI pipeline. Cryptic relatedness was modeled via the derivation of genomic relatedness matrices.

**Supplementary Method** **S4.** Additional details on the ISGC summary statistics used in the mtCOJO, genetic correlation and GSMR analysis

Cohorts included in the ISGC summary statistics for suicidal attempt phenotype for mtCOJO, genetic correlation and GSMR analysis are listed below. For this meta-analysis, University of Utah, Japan, and Columbia (because 50% of those cases had died by suicide) were excluded in the meta-analysis but samples from all ancestry were included.

| Dataset | N_cases | N_controls | LAMBDA-GC | N-SNPs | N_eff_half |
| --- | --- | --- | --- | --- | --- |
| PGC_BIP | 3214 | 17642 | 1.031 | 7442691 | 5437 |
| PGC_ED | 170 | 5070 | 0.983 | 5587543 | 328 |
| PGC_MDD | 1528 | 16626 | 1.011 | 7016830 | 2798 |
| PGC_SCZ | 1640 | 7112 | 1.016 | 7991663 | 2665 |
| AGDS | 2792 | 20193 | 1.025 | 7597490 | 4905 |
| ArmySTARRS | 670 | 10637 | 1.01 | 8883722 | 1260 |
| Borderline | 481 | 1653 | 1.029 | 7478057 | 745 |
| GISS | 660 | 660 | 0.993 | 6807933 | 660 |
| iPsych | 7003 | 52227 | 1.068 | 9470354 | 12350 |
| Janssen | 255 | 1684 | 1.019 | 6905784 | 442 |
| UKB | 2433 | 334766 | 1.037 | 7776903 | 4830 |
| YalePenn_EA | 475 | 1817 | 1.03 | 6036297 | 753 |
| CONVERGE | 1148 | 6515 | 1.015 | 6708328 | 1952 |
| GTP | 669 | 4473 | 0.993 | 15853283 | 1163 |
| YalePenn_AA | 629 | 2902 | 1.035 | 14490501 | 1033 |
| SUM | 23767 | 483977 | 1.14 | 7780128 | 41328 |

**Supplementary Method** **S5.** Cross-reference of this study results with other published GWAS results

To check for potential supporting evidence from this study to published GWAS results, a list of variants compiled in the Supplementary Table of a previously published GWAS^2^, as well as the recently published ISGC GWAS meta-analysis^4^ with a p-value less than 5 x 10^-6^, were used, a finding was considered significant if it passed multiple testing correction thresholds. All results with a nominal p-value less than 0.05 are also reported.

**Supplementary Method** **S6.** Additional details on the summary statistics used in the PRS association analysis

Traits for calculating PRS included psychiatry, somatic comorbidity traits, personality traits, lifestyle factors. A full list of PRSs derived are available in Supplementary Table S2 and these included suicidal attempt^38^, depression^39, 40^, bipolar disorder^30^, schizophrenia^23^, attention-deficit/hyperactivity disorder (ADHD)^41^, autism spectrum disorder (ASD)^42^, posttraumatic stress disorder (PTSD)^43^, anxiety and stress-related disorders^44, 45^, cannabis use disorder^46^, tobacco and alcohol use^47^, educational attainment (EA) and cognitive performance^48^, intelligence^49^, general cognitive function^50^, insomnia^51^, neuroticism^40^, pain^52, 53^, subjective well-being^54^, human subcortical brain structures^55^, risk tolerance and risk behaviors including drinking and smoking^56^, C-reactive protein^57^, blood lipids^58, 59^, coronary artery disease (CAD)^60^, and anthropometric traits such as waist-to-hip ratio^61^ and body mass index (BMI)^62^, etc.

**Supplementary Method** **S7.** Additional details on the summary statistics used in the genetic correlation analysis

Traits for calculating genetic correlation included depression^39^, bipolar disorder^31^, schizophrenia, ADHD^41^, ASD^42^, PTSD^43^, anxiety disorders^44^, panic disorder^63^, EA^48^, insomnia^51^, neuroticism^40^, chronic pain^52^, risk tolerance and risk behaviors^56^, and body mass index (BMI)^62^.

**Supplementary Text S1**. Replication results

Our suicide death study replicated the genome-wide significant finding in rs116955121^64^ that was associated with suicidal ideation and attempt in UKB after adjusting for psychiatric condition (*p* = 0.0003 in our SD GWAS meta-analysis, Supplementary Table S6). Accounting for 51 independent suggestive association loci from the ISGC meta-analysis (Supplementary Table S7), no marker was replicated using the SD GWAS meta-analysis. Nominal replication (p < 0.05) is however provided in Supplementary Table S8.

For our suicidal behavior results, we also provide “replication” evidence for one out of fifty-one genomic regions with suggestive association evidence (*p* < 5 x 10^-6^) from the ISGC meta-analysis (Supplementary Table S14), which was mapped to *SDK1* in chromosome 7. If using FinnGen cohort results alone, which is based on a completely independent sample from the ISGC samples, we were only able to nominally replicate a genomic locus in chromosome 2 (*p* = 0.02) encompassing 11 genes including *CLK1* and *TRAK2* (Supplementary Tables S7 and S15).

**Supplementary Text S2.** Genetic correlation results for suicidal attempt using ISGC summary statistics (excluding death cohorts).

Suicidal attempt was additionally correlated with bipolar disorder (r_g_ = 0.57, *p* = 1.18 x 10^-48^), lifetime smoking status (r_g_ = 0.48, *p* = 8.35 x 10^-47^), schizophrenia (r_g_ = 0.43, *p* = 1.05 x 10^-33^), pain (r_g_ = 0.43, *p* = 1.24 x 10^-30^), risky behaviors captured by principal component 1 of four risky behaviors (also known as disinhibition) (r_g_ = 0.39, *p* = 1.15 x 10^-25^), ADHD (r_g_ = 0.52, *p* = 5.65 x 10^-25^), risk tolerance (r_g_ = 0.33, *p* = 8.14 x 10^-16^), insomnia (r_g_ = 0.33, *p* = 1.36 x 10^-14^), PTSD (r_g_ = 0.71, *p* = 6.56 x 10^-14^), educational attainment (r_g_ = -0.22, *p* = 1.18 x 10^-12^), and neuroticism (r_g_ = 0.47, *p* = 1.48 x 10^-12^).

To examine whether these genetic correlations were mediated by depression, r_g_ was estimated with the same traits using the SA|MDD results. SA genetic correlations with depression, schizophrenia, bipolar disorder, ever smoker status, ADHD, pain, PTSD, risk tolerance, risky behaviors, and EA remained significant after conditioning, while the correlations for ASD, anxiety, insomnia, neuroticism, and BMI did not remain significant after conditioning.

**Supplementary Text S3**. Discussions on suggestive associations from gene-based association analysis.

A few genes with gene-level suggestive association evidence across multiple analyses also merit comment. *LIMK2* is involved in neurodevelopment, neuronal plasticity, and dendritic spine remodeling. The top differentially methylated position identified in an epigenome-wide association study comparing first-episode schizophrenia patients and controls in a Han Chinese cohort was annotated to *LIMK2*^65^. *LIMK1* and *LIMK2* transcripts were also upregulated in laminar and cellular samples, while *CDC42* transcript was downregulated in pyramidal cells in micro-dissected dorsolateral prefrontal cortical layer 3 samples from patients with schizophrenia, supporting the role of the *CDC42* pathway in destabilizing actin dynamics and resulting spine deficits in schizophrenia^66^. A chromosome 11 suggestive SD association signal (lead variant rs3741042, *p* = 2.68 x 10^-6^) also implicated *EIF4G2* via chromatin interaction in fetal cortex. Network analysis identified "GNA13-ERK1-eIF4G2 signaling" as a downregulated network, and proteins involved in this network were significantly decreased in the prefrontal cortex of schizophrenia patients^67^. eIF4A1/2 and CYFIP1 downstream of eIF4G2 were also decreased, suggesting that down-regulation of the network suppresses expression of *CYFIP1* that play a role in actin remodeling, and is involved in axon outgrowth and spine formation^67^. These processes are critical for neuronal plasticity and dendritic spine remodeling, and the involvement of *LIMK2* and *CDC42* was confirmed in laser-captured serotonin neurons from *rhesus macaques* upon hormone challenge^68^. Finally, an *NRBF2* rare variant contribution was implicated in a bipolar disorder whole-exome sequencing and linkage analysis^69^.

**Supplementary Figure 1.** Step by step QC for SD cohorts using the RICOPILI pipeline^33^. Please also refer to https://sites.google.com/a/broadinstitute.org/ricopili and http://bit.ly/2ZfBNJj for additional details of the RICOPILI pipeline and QC report. Iterative process of case control matching in cohort 1 A-C and cohort 2 D-H and the resulting final samples in SD GWAS meta-analysis [Cohort 1 & 2 (I)].

1. **Cohort 1 Round 1 QC**

1. **Cohort 1 Round 2 QC**

1. **Cohort 1 Round 3**

1. **Cohort 2 Round 1**

1. **Cohort 2 Round 2**

**Sample size in QC prior to PCA analysis:**

1. **Cohort 2 Round 3**

**Sample size in QC prior to PCA analysis**

1. **Cohort 2 Round 4**

**Sample size in QC prior to PCA analysis**

1. **Cohort 2 Round 5 QC**

Note that the lambda used in the QC was based on logistic regression, and for GWAS meta-analysis, we used results from linear mixed model to control the lambda inflation perhaps due to the cryptic relatedness in the case samples

1. **SD death cohort 1 and 2 combined - final samples SD GWAS meta-analysis (further removing the overlapping samples between cohort 1 and 2)**

**Supplementary Figure 2** QQ-plots for suicide death GWAS meta-analysis: **SNP-level associations** - (A), suicide death condition on MDD (suicide death | MDD, B); and suicide death condition on suicidal attempt (suicide death | attempt, C) and **gene-level associations** - suicide death (D), suicide death | MDD (E); and suicide death | attempt (F); and suicidal behavior GWAS meta-analysis. **SNP-level associations** for suicidal behavior (G) and suicidal behavior condition on MDD (suicidal behavior | MDD, H), and **gene-level associations** for suicidal behavior (I) and suicidal behavior | MDD (J).

(A)


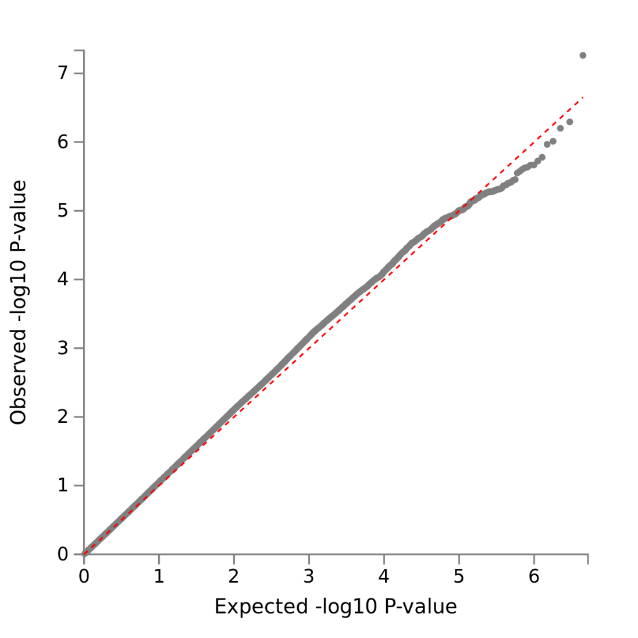


(B)


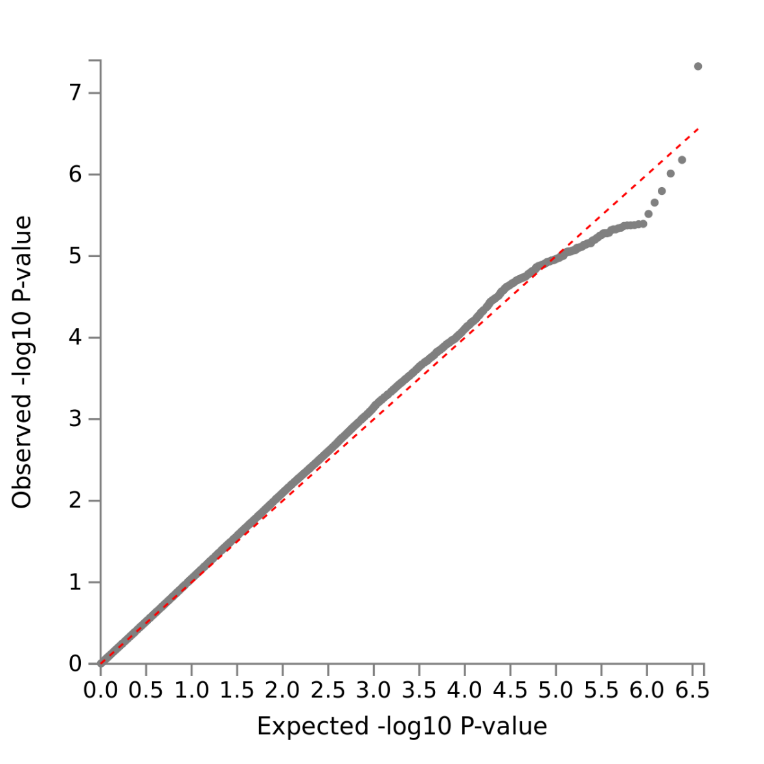


(C)


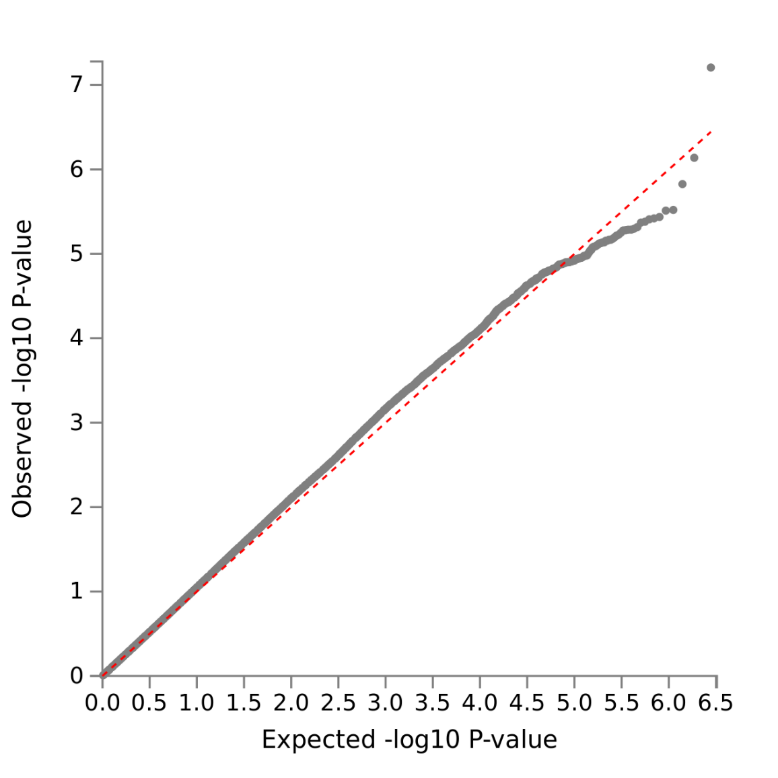


(D)


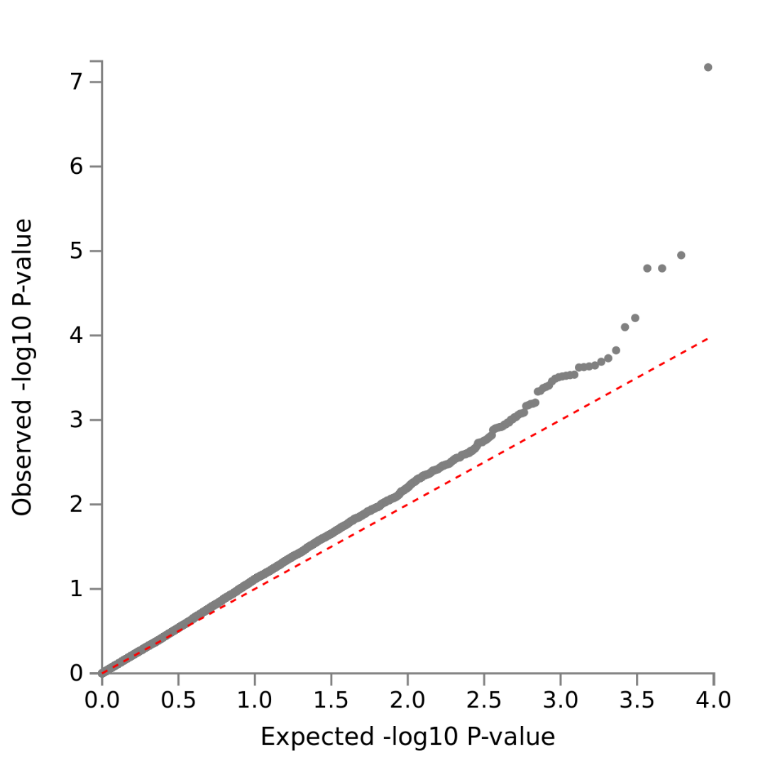


(E)


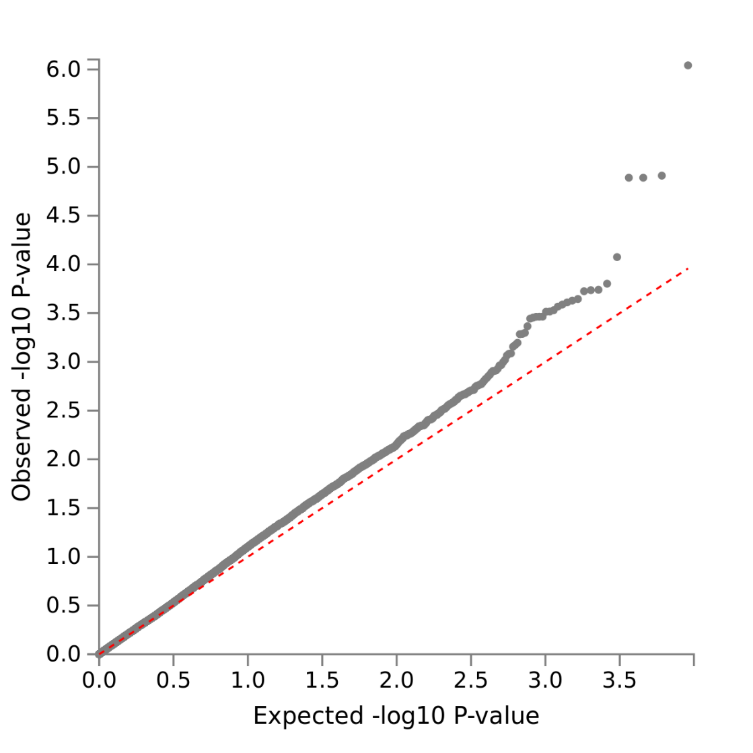


(F)


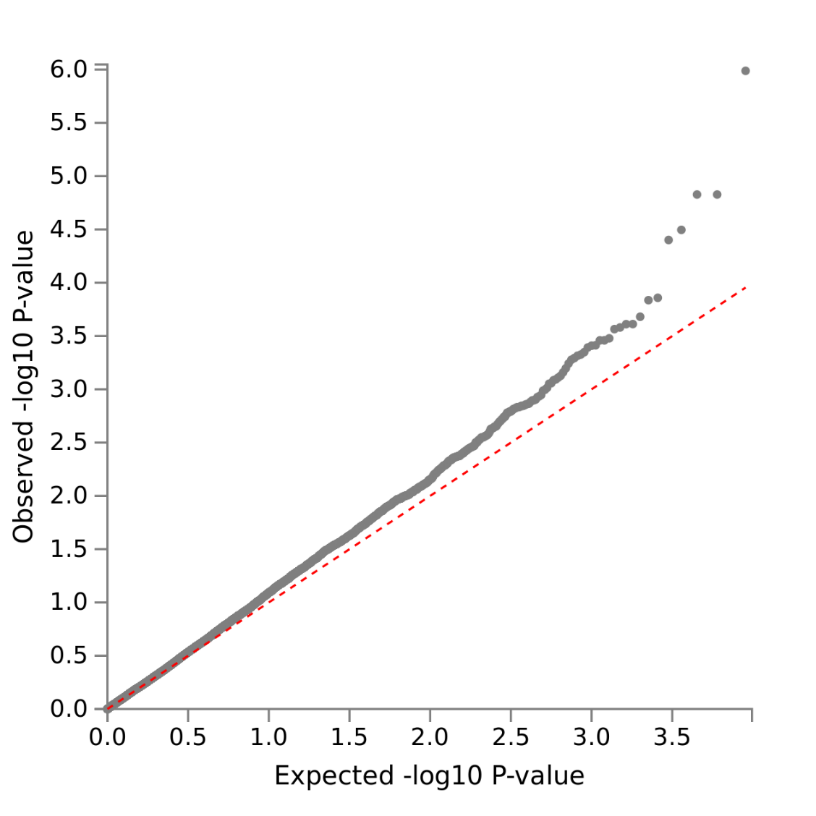


(G)


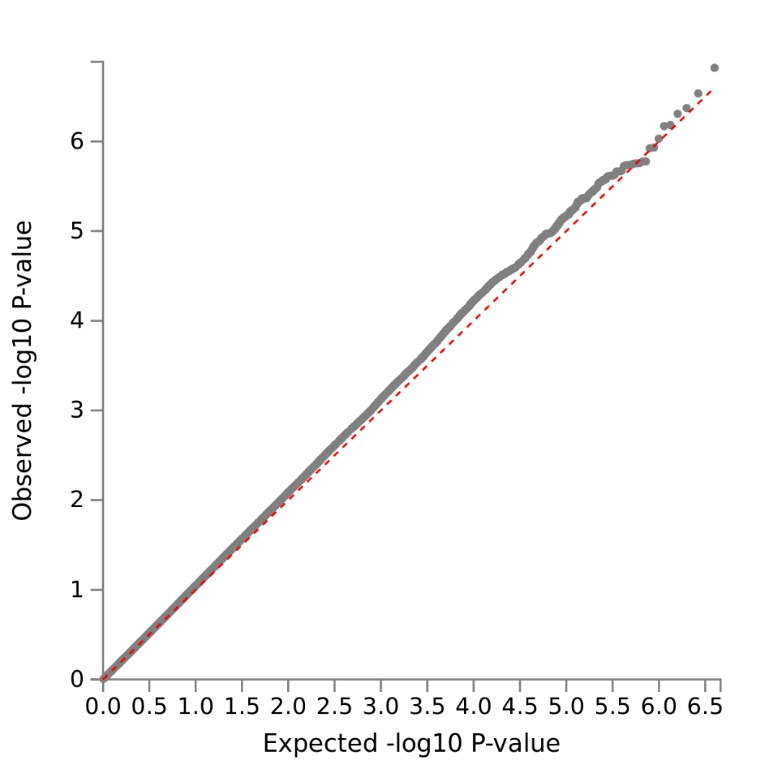


(H)


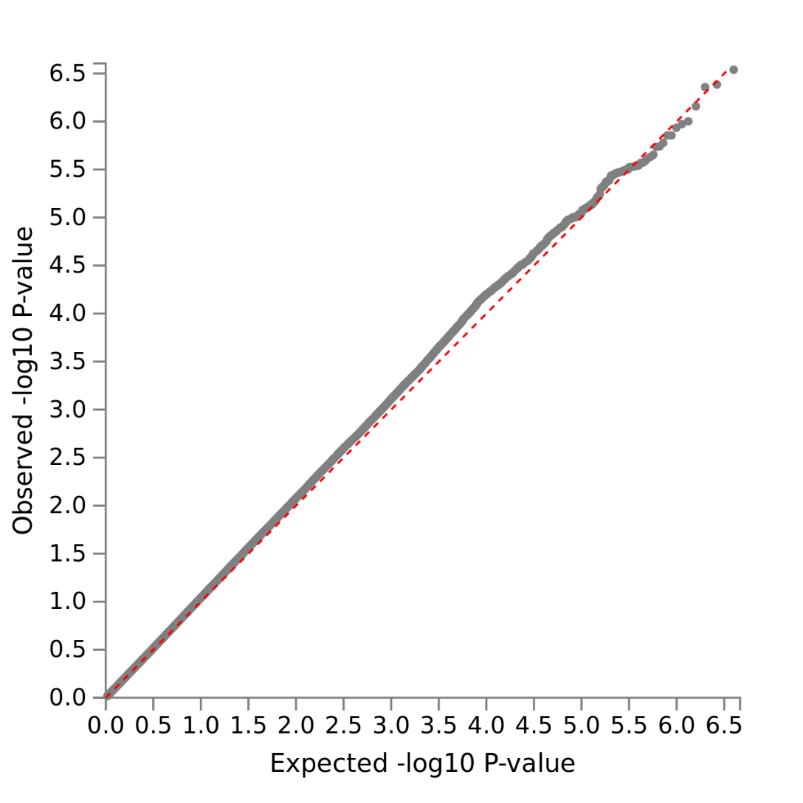


(I)


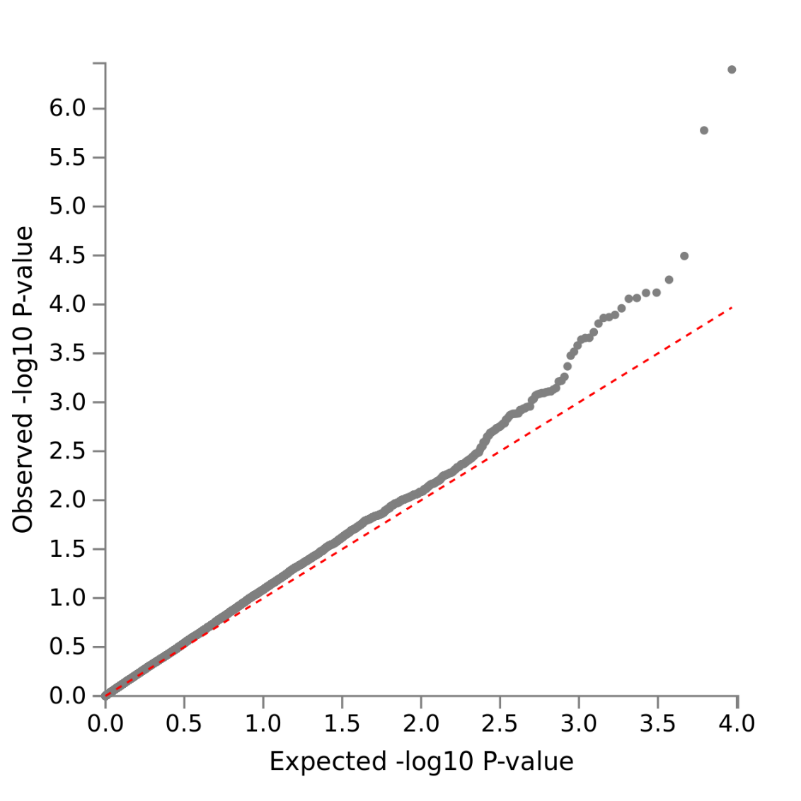


(J)


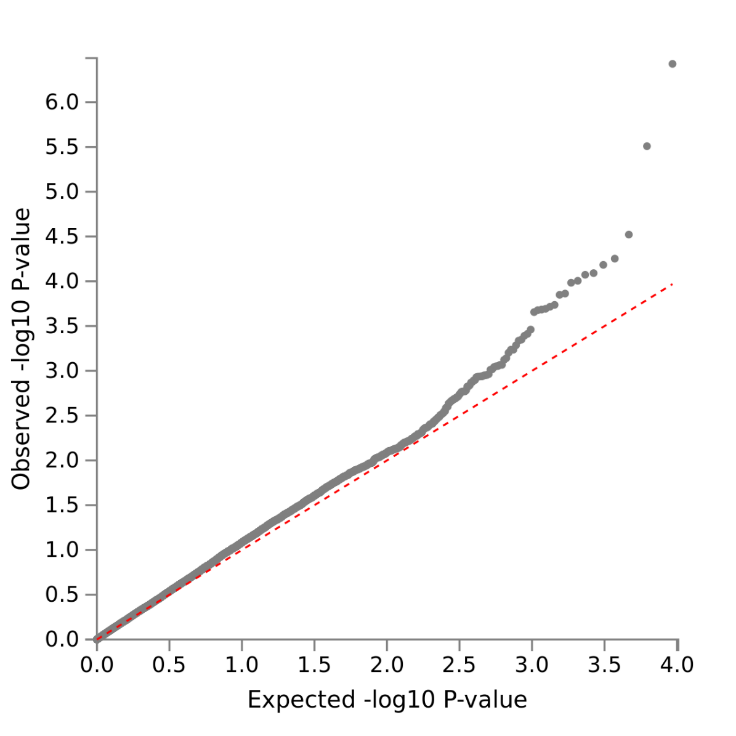


**Supplementary Figure 3** Genome-wide significant association signals. Manhattan plots for SNP-level associations for suicide death | MDD (A); and suicide death | attempt (B), and gene-level associations for suicide death | MDD (C); and suicide death | attempt (D); and for suicidal behavior | MDD: SNP-level associations (E) and gene-level associations (F).

(A)


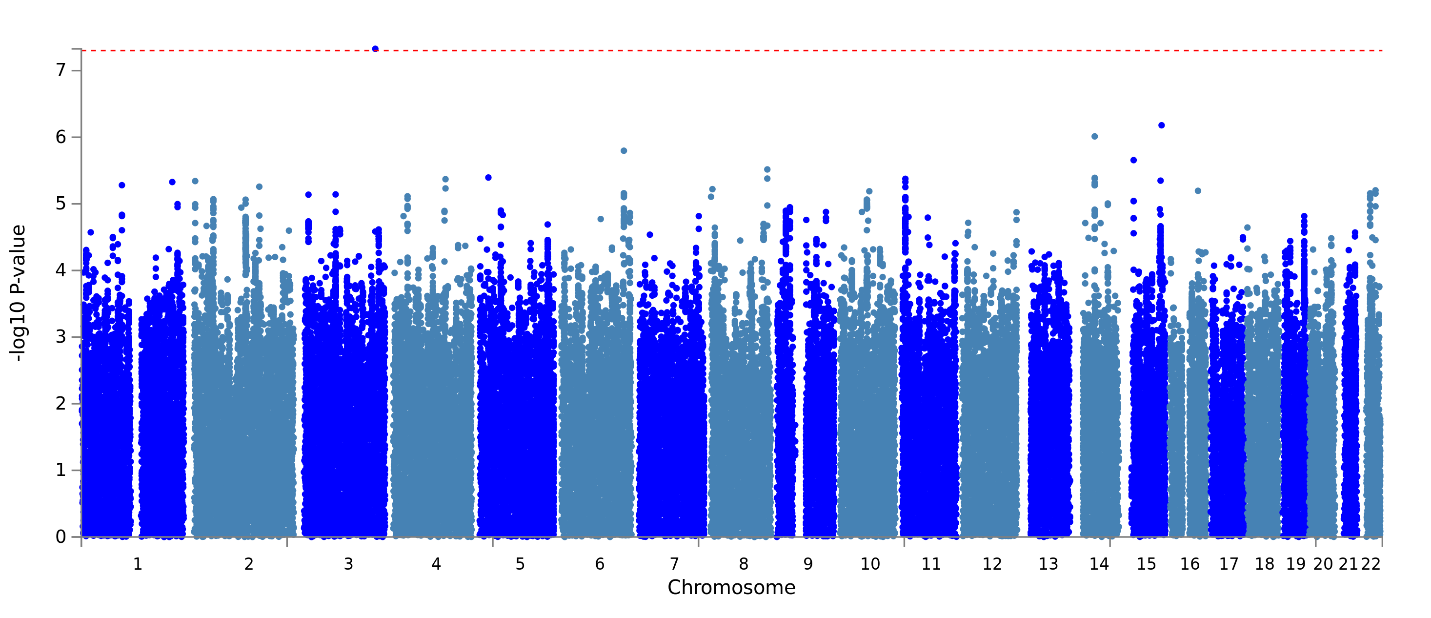


(B)


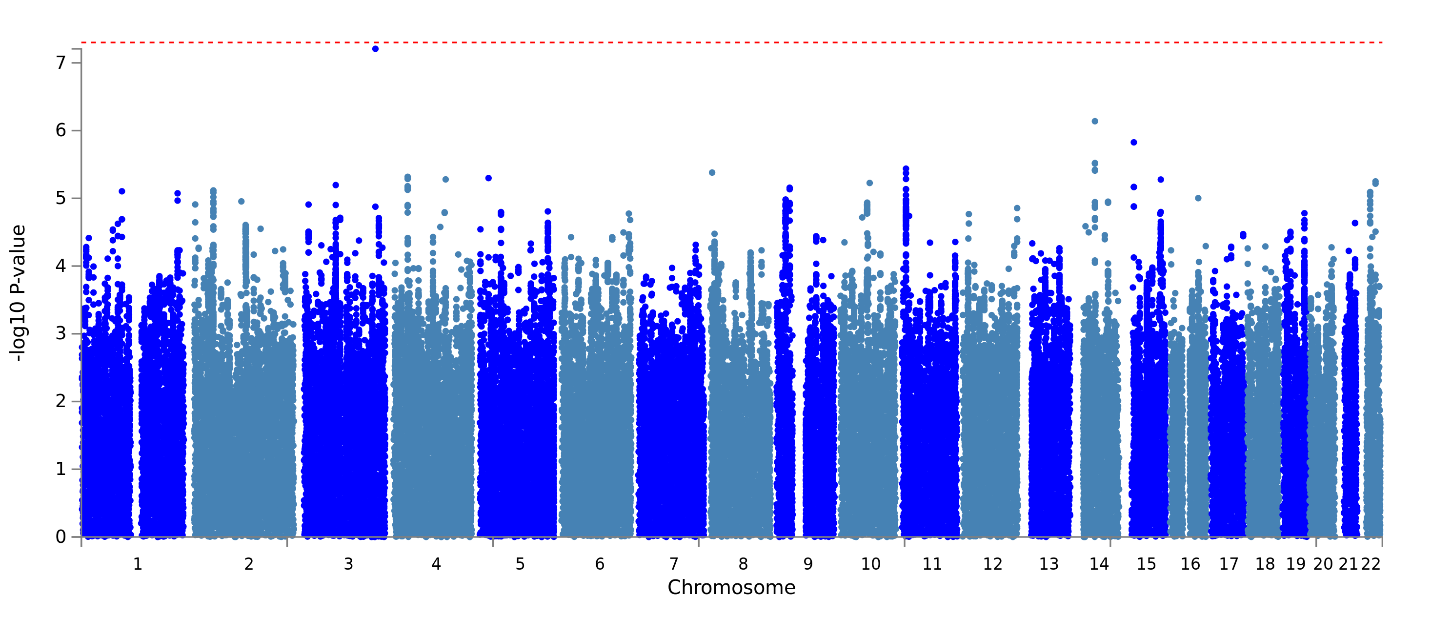


(C)

**
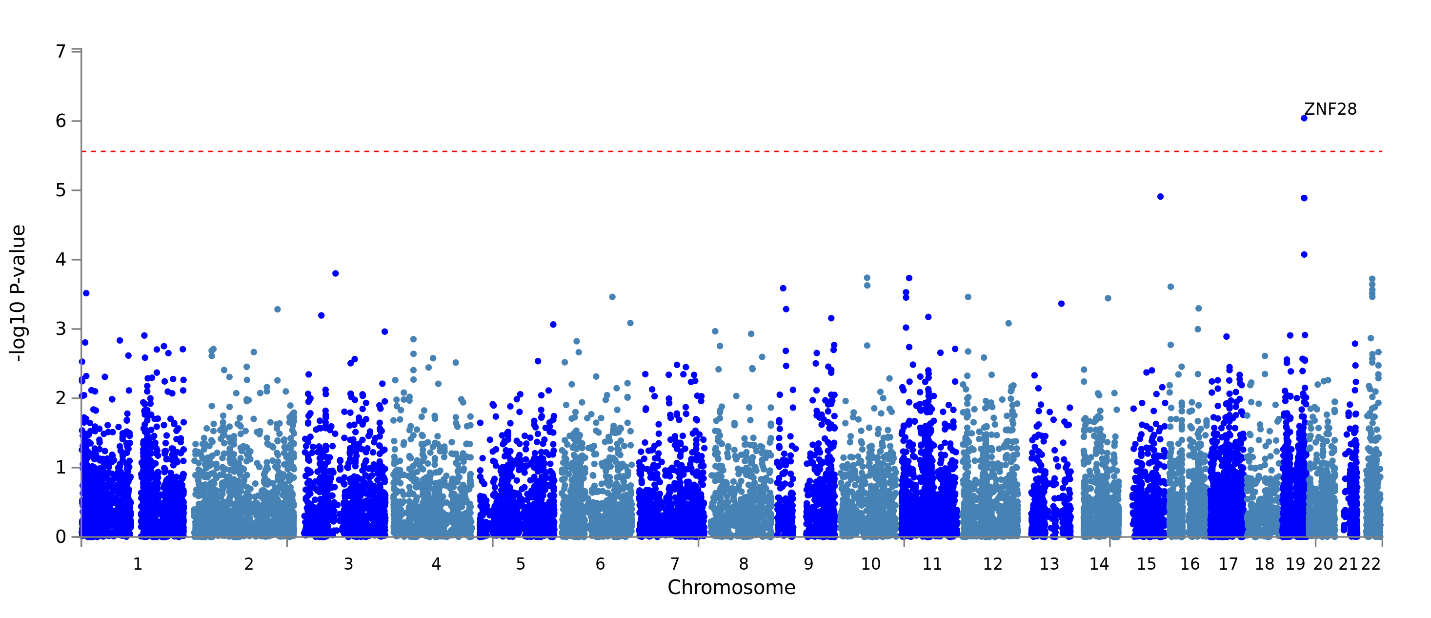
**

**(D)**

**
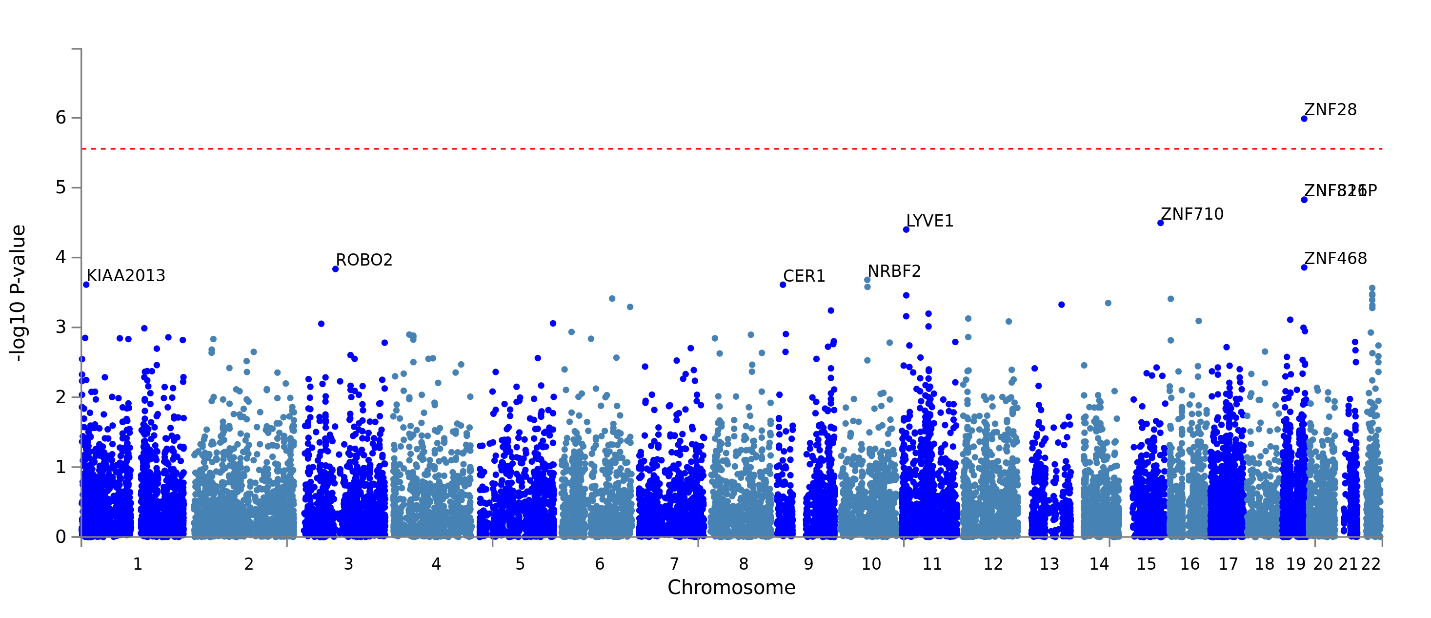
**

**(E)**

**
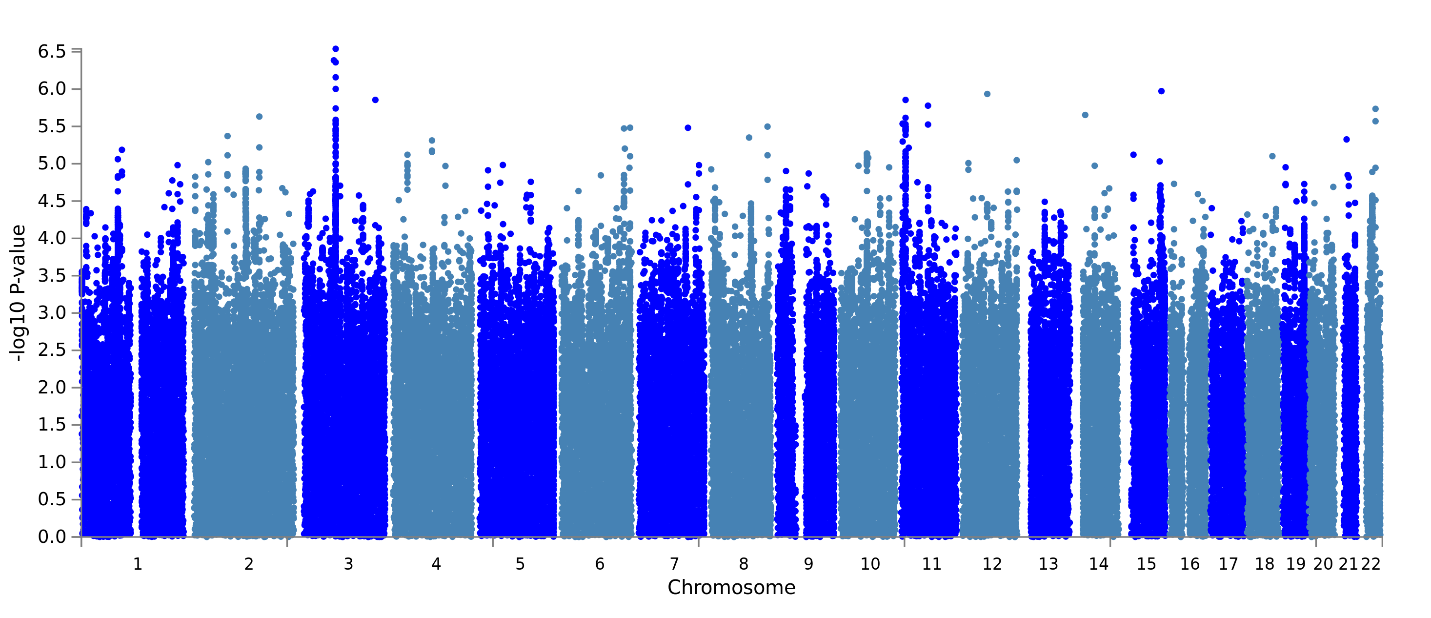
**

**(F)**

**
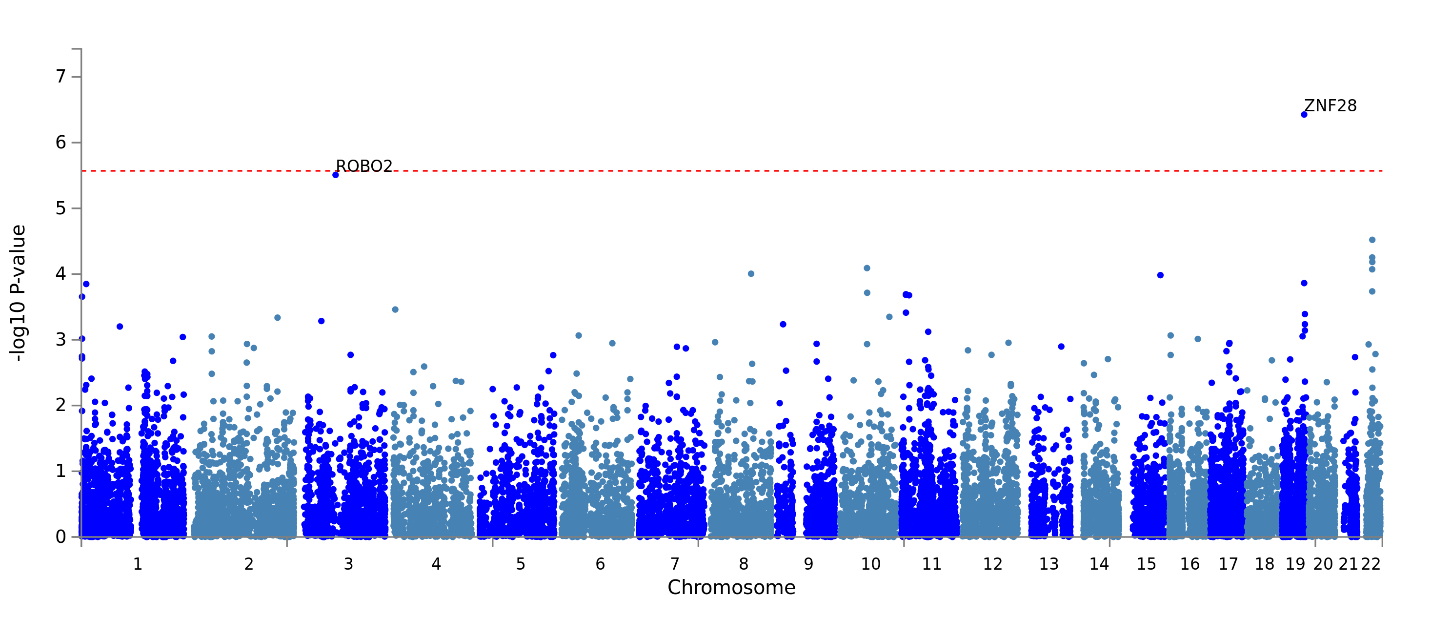
**

**Supplementary Figure 4** LocusZoom regional plots for additional loci for suicide death (A) 15: 93,058,525 G/A, rs9672835, near C15orf32 (B) 14: 48,538,317 A/G, rs1402514, near AL359212.1.

1. 15: 93,058,525 G/A, rs9672835, near C15orf32


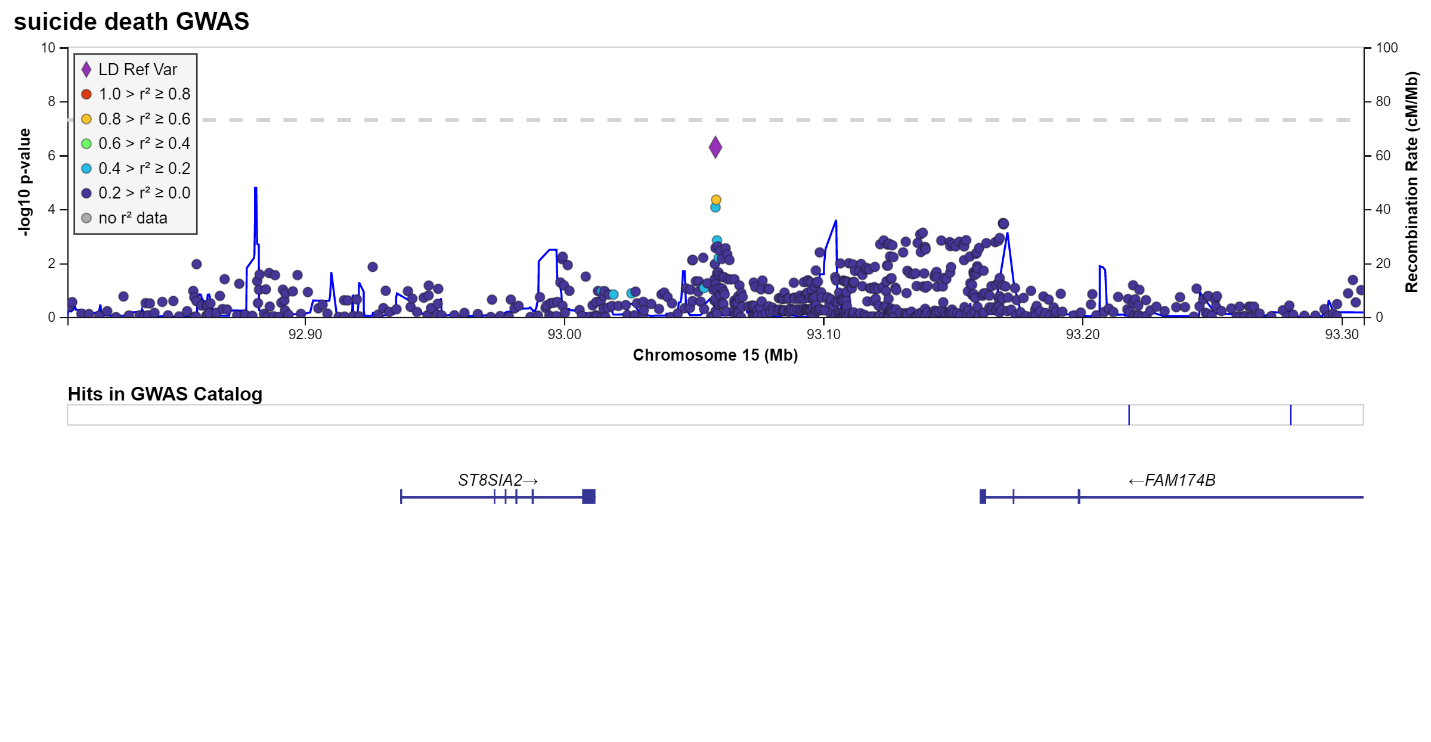


1. 14: 48,538,317 A/G, rs1402514, near AL359212.1.


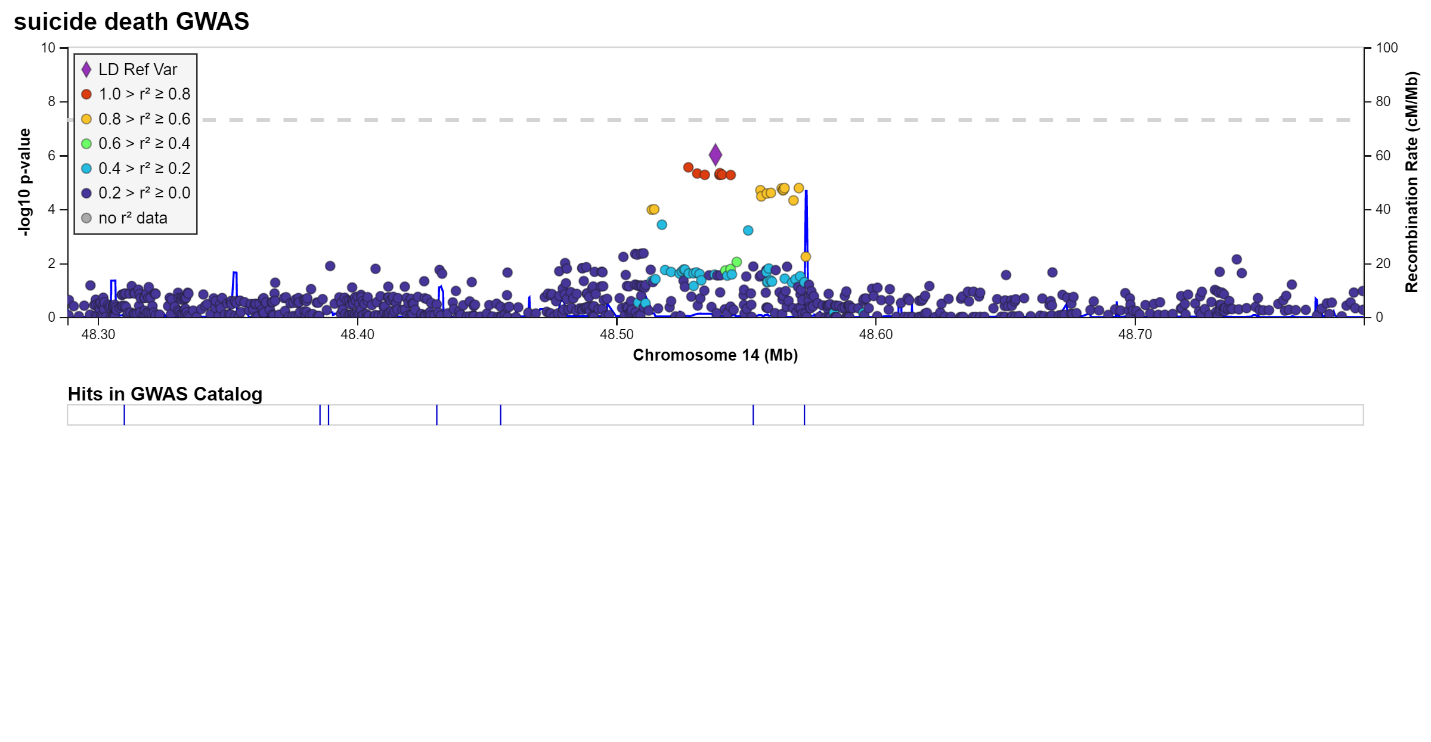


**Supplementary Figure 5** LocusZoom regional plots for additional loci for suicidal behavior (A) 3: 72,817,573 C/T rs9882095 near *SHQ1;* (B) 5: 23,048,728 T/C near *AC010445.1;* (C) 15: 93,058,525 G/A rs9672835 near *C15orf32*; (D) [3: 73,077,944 T/C](https://my.locuszoom.org/gwas/605308/region/?chrom=3&start=72827944&end=73327944) near *PPP4R2; (E)* [5: 120,289,227 G/A](https://my.locuszoom.org/gwas/605308/region/?chrom=5&start=120039227&end=120539227) near *AC008565.1; (F)* [3: 73,077,944 T/C](https://my.locuszoom.org/gwas/605308/region/?chrom=3&start=72827944&end=73327944) near *PPP4R2; (G)* [3: 73,077,944 T/C](https://my.locuszoom.org/gwas/605308/region/?chrom=3&start=72827944&end=73327944) near *PPP4R2; (H)* [5: 120,289,227 G/A](https://my.locuszoom.org/gwas/605308/region/?chrom=5&start=120039227&end=120539227) near *AC008565.1.*

1. 3: 72,817,573 C/T rs9882095 near *SHQ1*


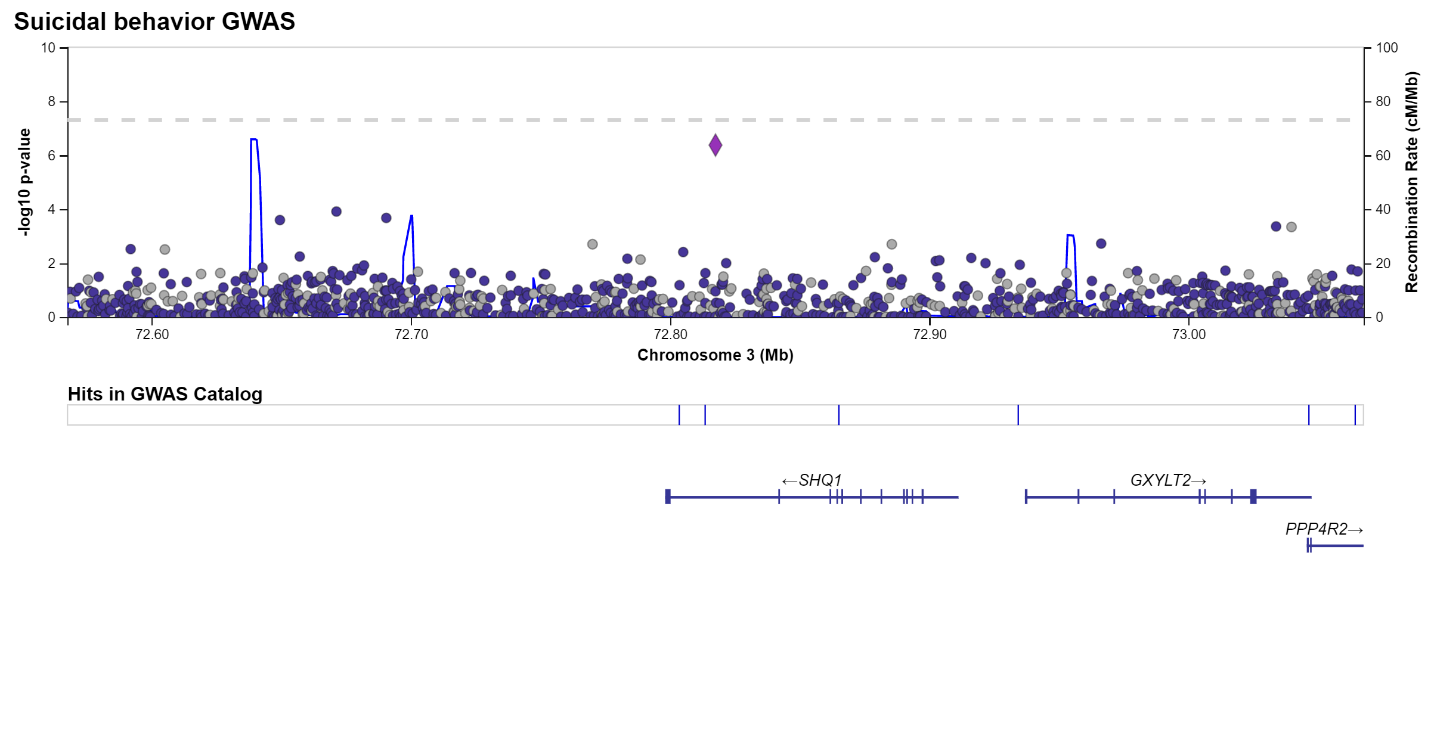


1. 5: 23,048,728 T/C near *AC010445.1*


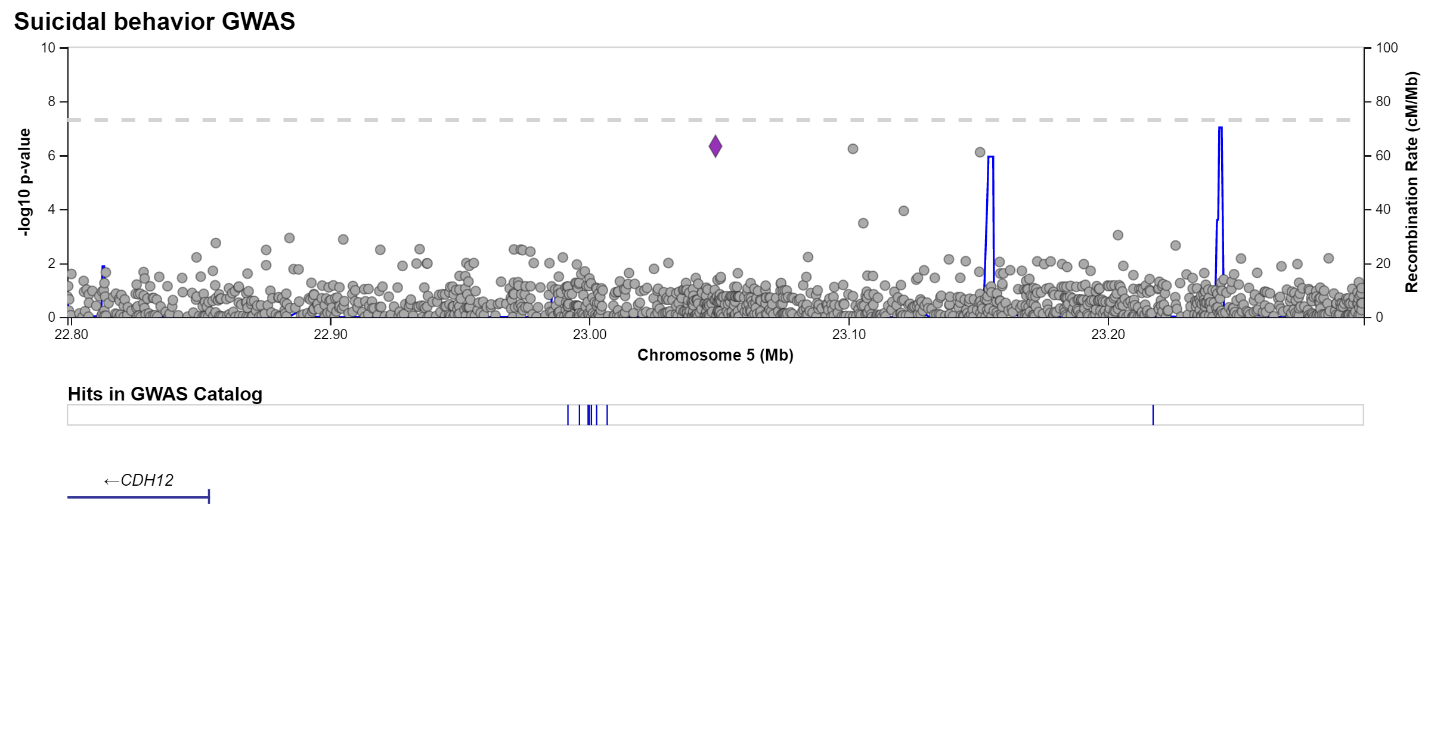


1. 15: 93,058,525 G/A rs9672835 near *C15orf32*


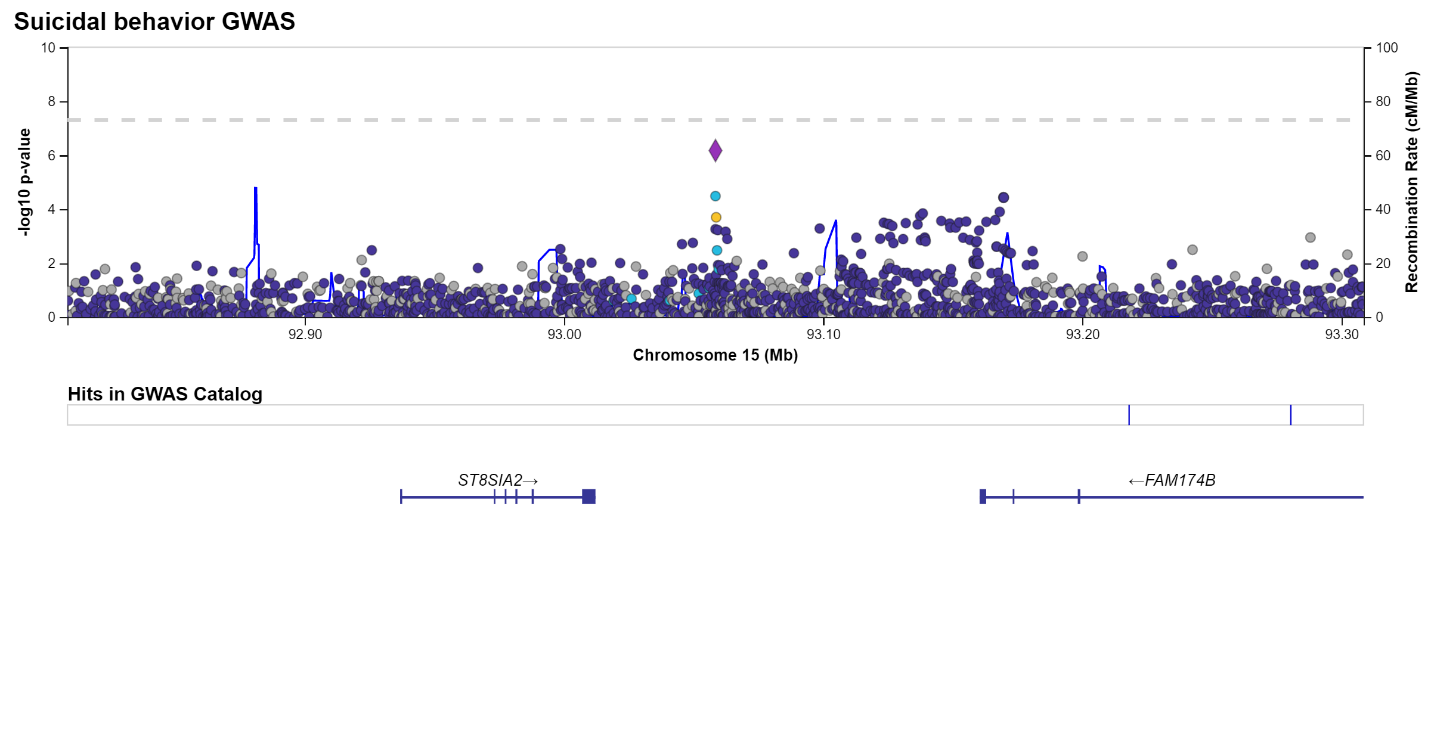


1. [3: 73,077,944 T/C](https://my.locuszoom.org/gwas/605308/region/?chrom=3&start=72827944&end=73327944) near *PPP4R2*


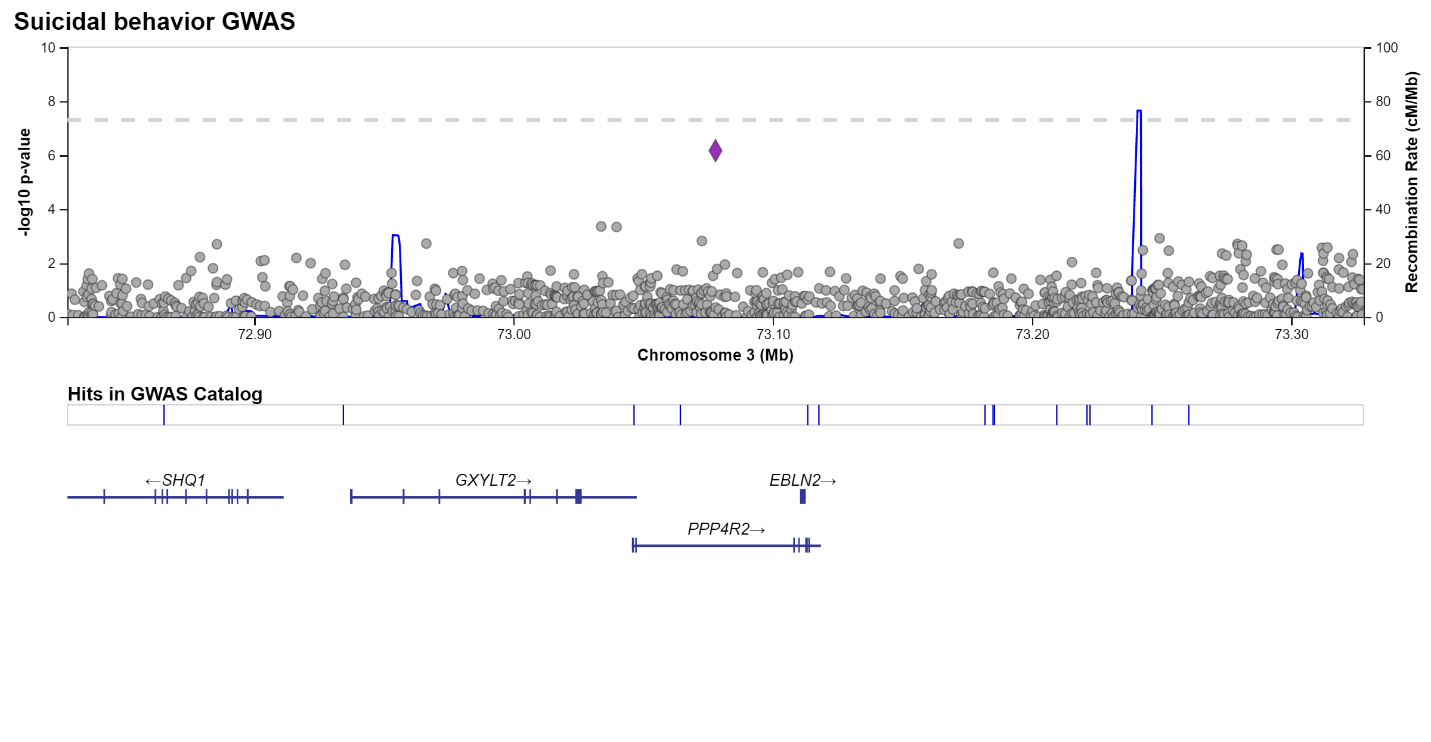


1. [5: 120,289,227 G/A](https://my.locuszoom.org/gwas/605308/region/?chrom=5&start=120039227&end=120539227) near *AC008565.1*


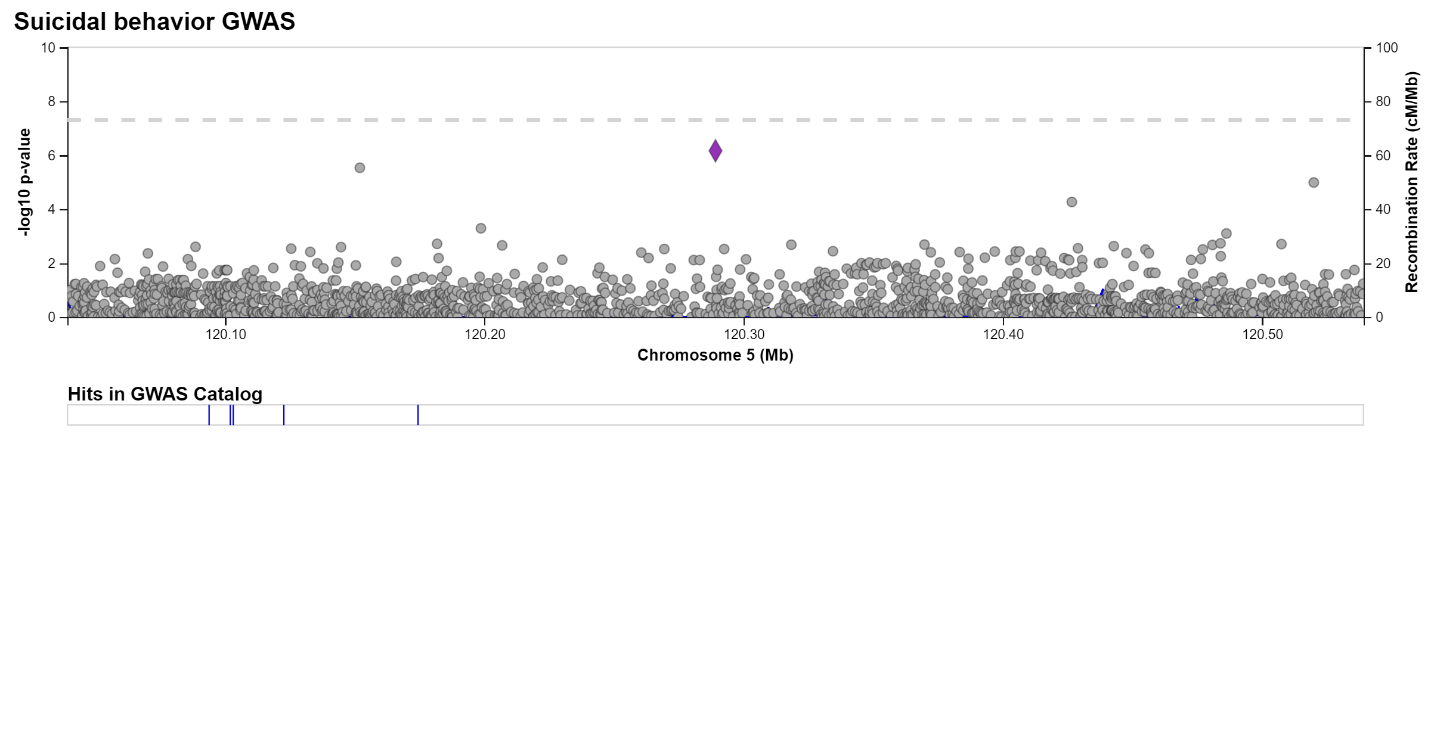


1. [2: 81,638,977 C/T](https://my.locuszoom.org/gwas/605308/region/?chrom=2&start=81388977&end=81888977) near *AC012075.1*


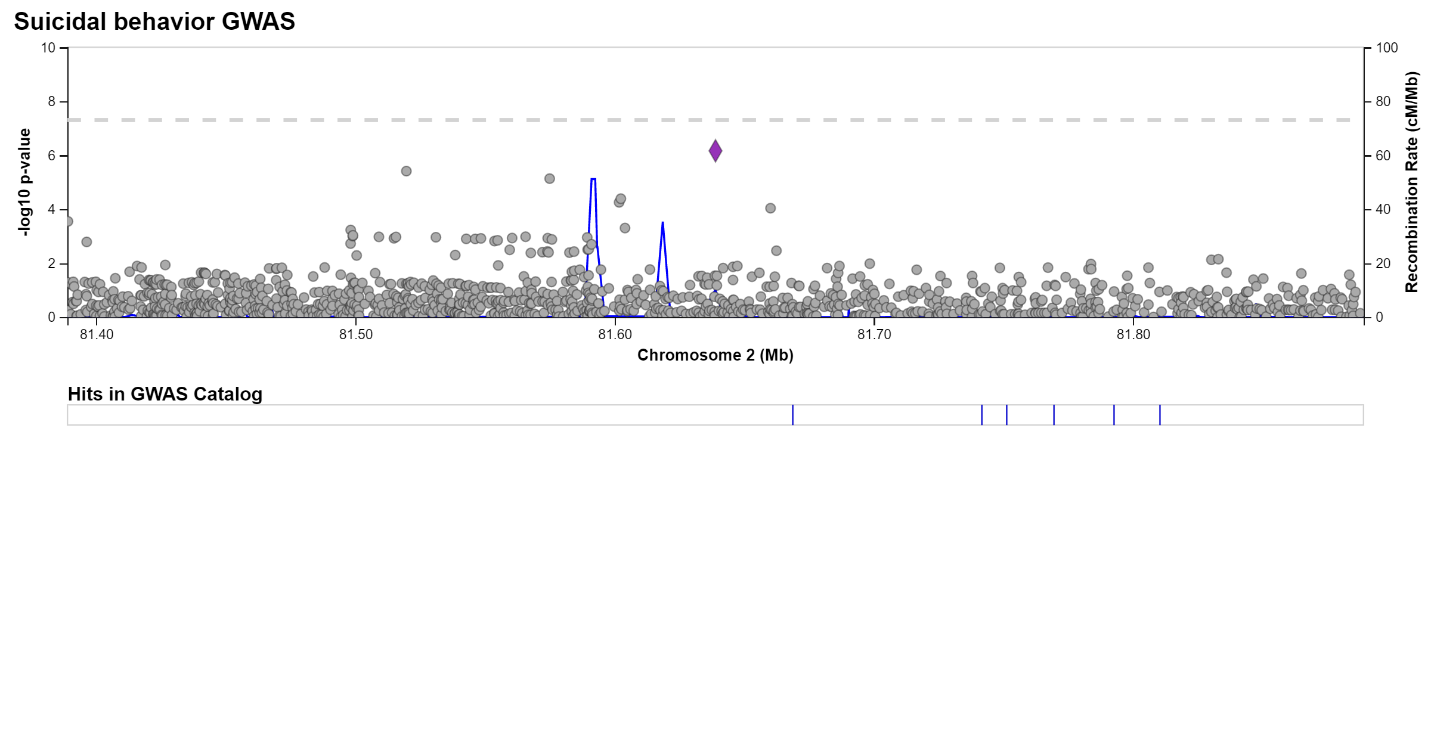


1. [11: 10,631,247 C/T](https://my.locuszoom.org/gwas/605308/region/?chrom=11&start=10381247&end=10881247) rs3741042 near *LYVE1, MRVI1*


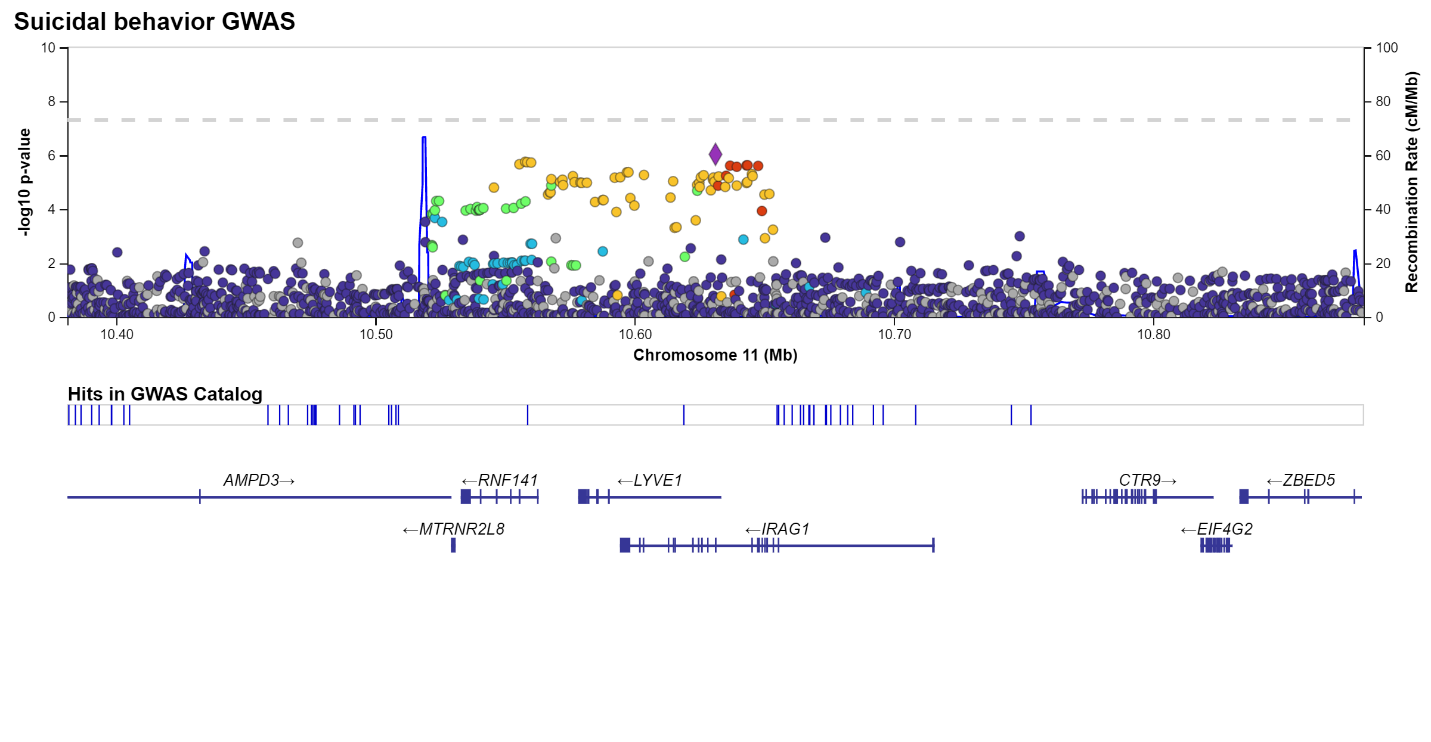


1. [9: 5,903,101 G/GTGT](https://my.locuszoom.org/gwas/605308/region/?chrom=9&start=5653101&end=6153101) near *KIAA2026, MLANA*


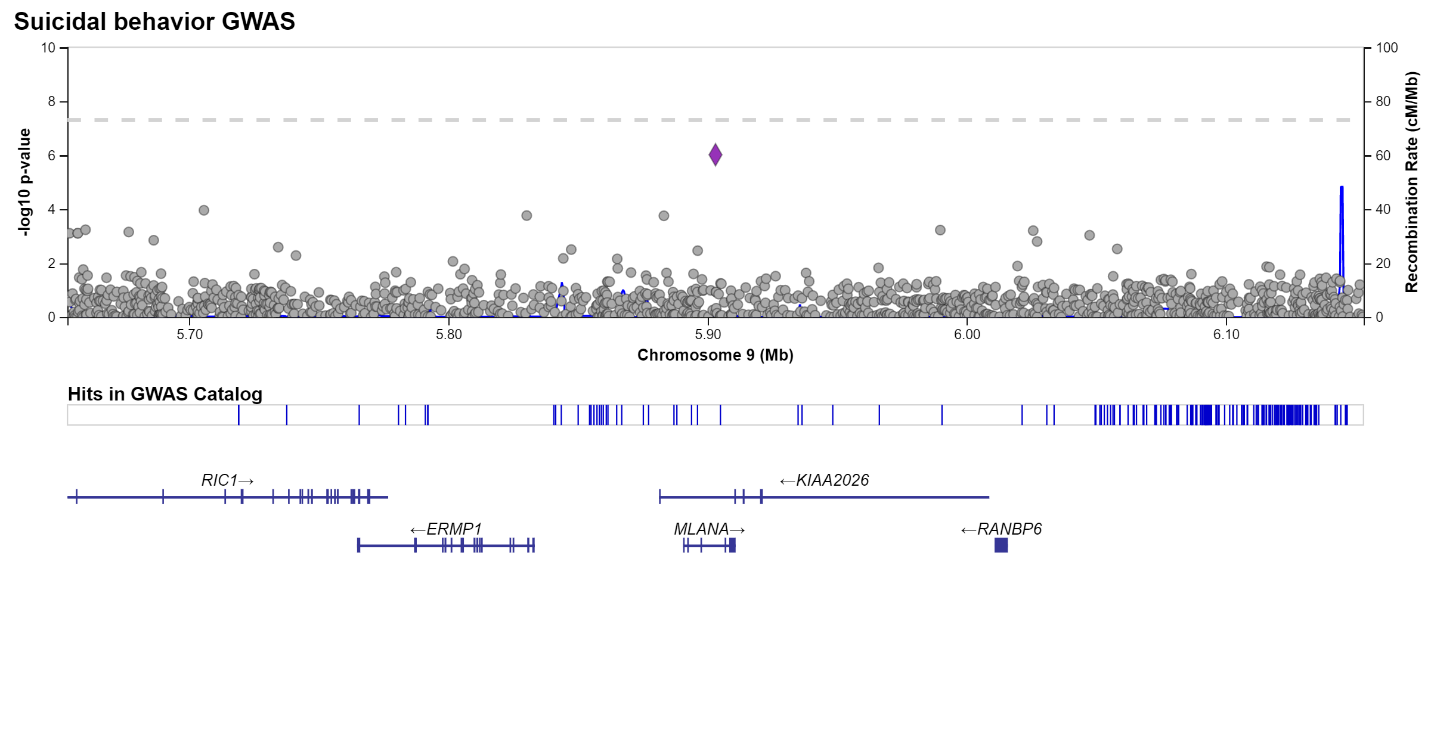


**Supplementary Figure 6.** GSMR analysis to test the bi-directional causal relationship between depression, suicidal attempt, and suicide death. SNP sets marked with * indicates a reduced stringency of SNP with p < 5 x 10^-5^ was used to select instrument variable for GSMR analysis, while those marked with ** indicates a p < 5x10^-6^ was used.


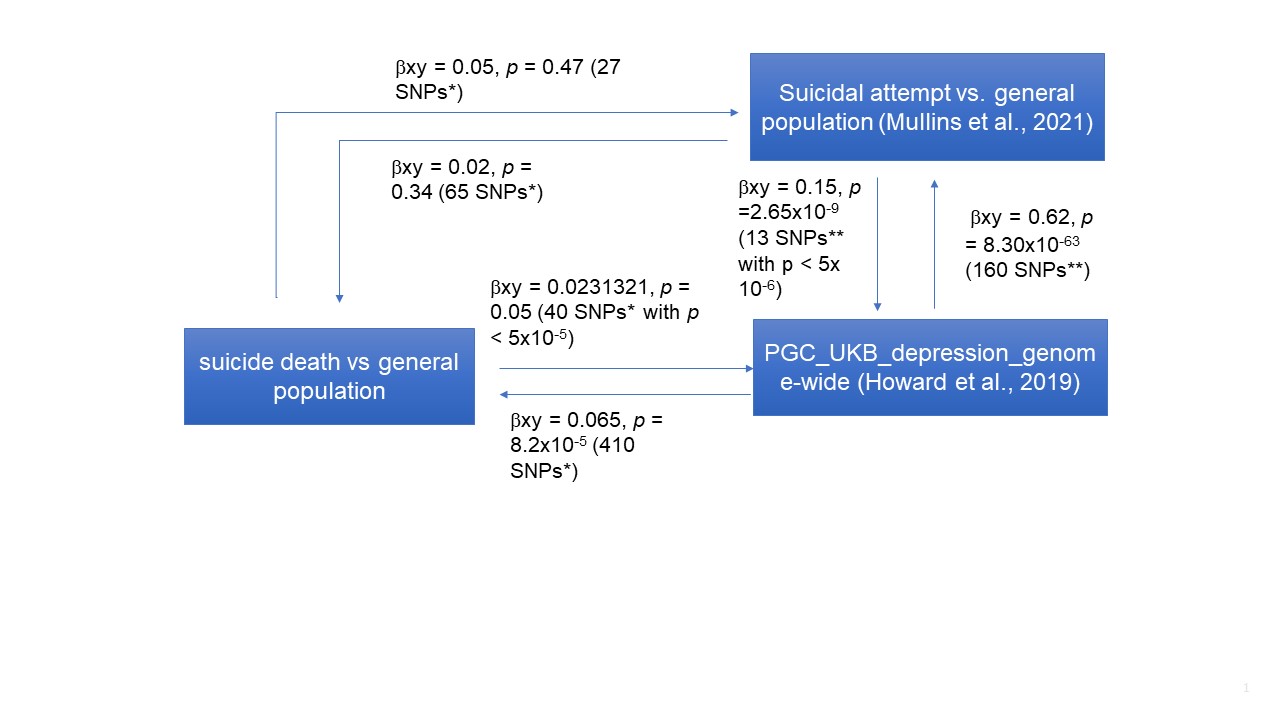


**Supplementary Figure 7** Expression level of *SOX5* from a reference RNA-Seq dataset [source: https://www.ncbi.nlm.nih.gov/gene/6660]


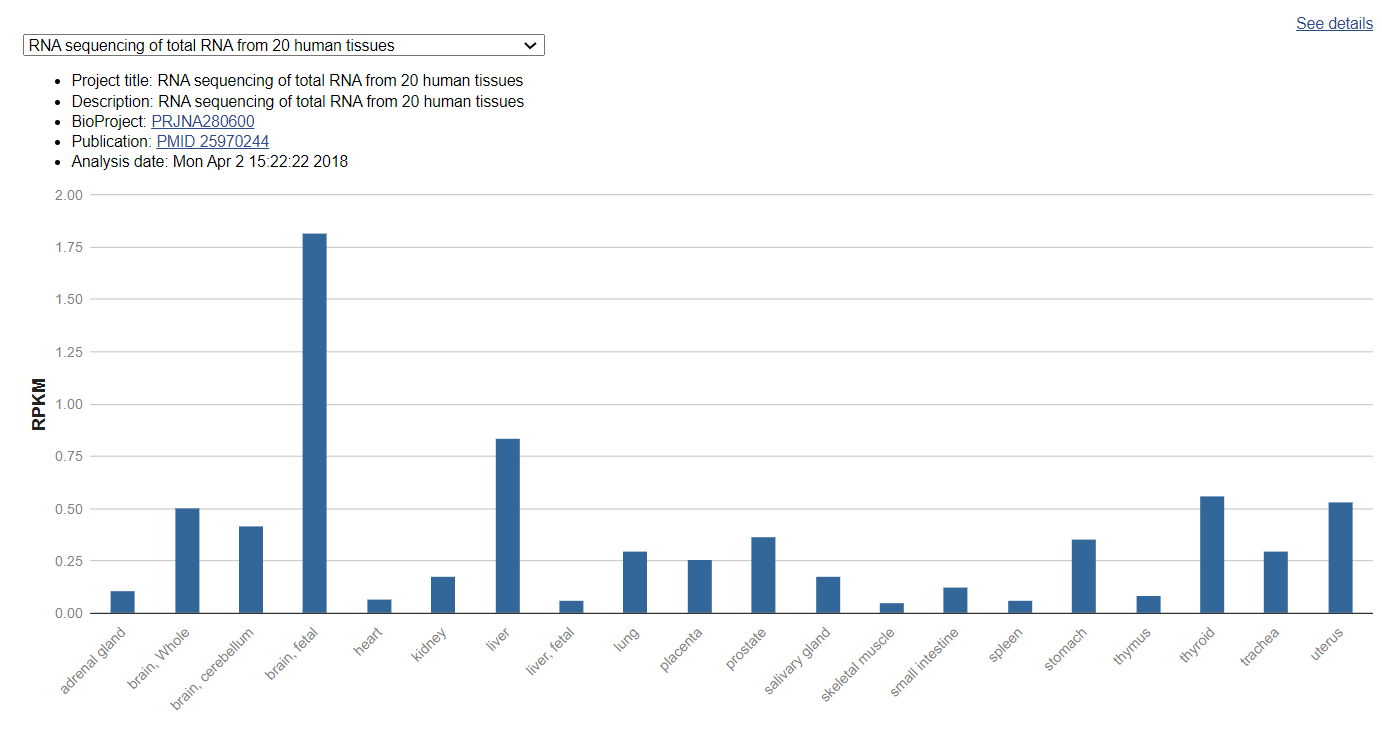


1. <https://le.utah.gov/xcode/Title26/Chapter4/C26-4_1800010118000101.pdf>, Accessed Date Accessed.

2. Docherty AR, Shabalin AA, DiBlasi E, Monson E, Mullins N, Adkins DE *et al.* Genome-Wide Association Study of Suicide Death and Polygenic Prediction of Clinical Antecedents. *The American journal of psychiatry* 2020; **177**(10)**:** 917-927.

3. Fritsche LG, Igl W, Bailey JN, Grassmann F, Sengupta S, Bragg-Gresham JL *et al.* A large genome-wide association study of age-related macular degeneration highlights contributions of rare and common variants. *Nature genetics* 2016; **48**(2)**:** 134-143.

4. Mullins N, Kang J, Campos AI, Coleman JRI, Edwards AC, Galfalvy H *et al.* Dissecting the Shared Genetic Architecture of Suicide Attempt, Psychiatric Disorders, and Known Risk Factors. *Biological psychiatry* 2021.

5. Ochs-Ross R, Daly EJ, Zhang Y, Lane R, Lim P, Morrison RL *et al.* Efficacy and Safety of Esketamine Nasal Spray Plus an Oral Antidepressant in Elderly Patients With Treatment-Resistant Depression-TRANSFORM-3. *Am J Geriatr Psychiatry* 2020; **28**(2)**:** 121-141.

6. Wajs E, Aluisio L, Holder R, Daly EJ, Lane R, Lim P *et al.* Esketamine Nasal Spray Plus Oral Antidepressant in Patients With Treatment-Resistant Depression: Assessment of Long-Term Safety in a Phase 3, Open-Label Study (SUSTAIN-2). *The Journal of clinical psychiatry* 2020; **81**(3).

7. Hirschfeld RM, Keck PE, Jr., Kramer M, Karcher K, Canuso C, Eerdekens M *et al.* Rapid antimanic effect of risperidone monotherapy: a 3-week multicenter, double-blind, placebo-controlled trial. *The American journal of psychiatry* 2004; **161**(6)**:** 1057-1065.

8. Smulevich AB, Khanna S, Eerdekens M, Karcher K, Kramer M, Grossman F. Acute and continuation risperidone monotherapy in bipolar mania: a 3-week placebo-controlled trial followed by a 9-week double-blind trial of risperidone and haloperidol. *Eur Neuropsychopharmacol* 2005; **15**(1)**:** 75-84.

9. Rapaport MH, Gharabawi GM, Canuso CM, Mahmoud RA, Keller MB, Bossie CA *et al.* Effects of risperidone augmentation in patients with treatment-resistant depression: Results of open-label treatment followed by double-blind continuation. *Neuropsychopharmacology : official publication of the American College of Neuropsychopharmacology* 2006; **31**(11)**:** 2505-2513.

10. Canuso CM, Dirks B, Carothers J, Kosik-Gonzalez C, Bossie CA, Zhu Y *et al.* Randomized, double-blind, placebo-controlled study of paliperidone extended-release and quetiapine in inpatients with recently exacerbated schizophrenia. *The American journal of psychiatry* 2009; **166**(6)**:** 691-701.

11. Macfadden W, Alphs L, Haskins JT, Turner N, Turkoz I, Bossie C *et al.* A randomized, double-blind, placebo-controlled study of maintenance treatment with adjunctive risperidone long-acting therapy in patients with bipolar I disorder who relapse frequently. *Bipolar Disord* 2009; **11**(8)**:** 827-839.

12. Canuso CM, Lindenmayer JP, Kosik-Gonzalez C, Turkoz I, Carothers J, Bossie CA *et al.* A randomized, double-blind, placebo-controlled study of 2 dose ranges of paliperidone extended-release in the treatment of subjects with schizoaffective disorder. *The Journal of clinical psychiatry* 2010; **71**(5)**:** 587-598.

13. Canuso CM, Schooler N, Carothers J, Turkoz I, Kosik-Gonzalez C, Bossie CA *et al.* Paliperidone extended-release in schizoaffective disorder: a randomized, controlled study comparing a flexible dose with placebo in patients treated with and without antidepressants and/or mood stabilizers. *J Clin Psychopharmacol* 2010; **30**(5)**:** 487-495.

14. Canuso CM, Turkoz I, Fu DJ, Bossie CA. Role of paliperidone extended-release in treatment of schizoaffective disorder. *Neuropsychiatr Dis Treat* 2010; **6:** 667-679.

15. Berwaerts J, Liu Y, Gopal S, Nuamah I, Xu H, Savitz A *et al.* Efficacy and Safety of the 3-Month Formulation of Paliperidone Palmitate vs Placebo for Relapse Prevention of Schizophrenia: A Randomized Clinical Trial. *JAMA Psychiatry* 2015; **72**(8)**:** 830-839.

16. Fu DJ, Turkoz I, Simonson RB, Walling DP, Schooler NR, Lindenmayer JP *et al.* Paliperidone palmitate once-monthly reduces risk of relapse of psychotic, depressive, and manic symptoms and maintains functioning in a double-blind, randomized study of schizoaffective disorder. *The Journal of clinical psychiatry* 2015; **76**(3)**:** 253-262.

17. Alphs L, Fu DJ, Turkoz I. Paliperidone for the treatment of schizoaffective disorder. *Expert Opin Pharmacother* 2016; **17**(6)**:** 871-883.

18. Bossie CA, Turkoz I, Alphs L, Mahalchick L, Fu DJ. Paliperidone Palmitate Once-Monthly Treatment in Recent Onset and Chronic Illness Patients With Schizoaffective Disorder. *J Nerv Ment Dis* 2017; **205**(4)**:** 324-328.

19. Fu DJ, Turkoz I, Walling D, Lindenmayer JP, Schooler NR, Alphs L. Paliperidone palmitate once-monthly maintains improvement in functioning domains of the Personal and Social Performance scale compared with placebo in subjects with schizoaffective disorder. *Schizophr Res* 2018; **192:** 185-193.

20. Savitz AJ, Xu H, Gopal S, Nuamah I, Mathews M, Soares B. Efficacy and safety of paliperidone palmitate 3-month formulation in Latin American patients with schizophrenia: A subgroup analysis of data from two large phase 3 randomized, double-blind studies. *Braz J Psychiatry* 2019; **41**(6)**:** 499-510.

21. Gopal S, Gogate J, Pungor K, Kim E, Singh A, Mathews M. Improvement of Negative Symptoms in Schizophrenia with Paliperidone Palmitate 1-Month and 3-Month Long-Acting Injectables: Results from a Phase 3 Non-Inferiority Study. *Neuropsychiatr Dis Treat* 2020; **16:** 681-690.

22. Johnson KM, Devine JM, Ho KF, Howard KA, Saretsky TL, Jamieson CA. Evidence to Support Montgomery-Asberg Depression Rating Scale Administration Every 24 Hours to Assess Rapid Onset of Treatment Response. *The Journal of clinical psychiatry* 2016; **77**(12)**:** 1681-1686.

23. Schizophrenia Working Group of the Psychiatric Genomics C. Biological insights from 108 schizophrenia-associated genetic loci. *Nature* 2014; **511**(7510)**:** 421-427.

24. Sleiman P, Wang D, Glessner J, Hadley D, Gur RE, Cohen N *et al.* GWAS meta analysis identifies TSNARE1 as a novel Schizophrenia / Bipolar susceptibility locus. *Scientific reports* 2013; **3:** 3075.

25. Li Q, Wineinger NE, Fu DJ, Libiger O, Alphs L, Savitz A *et al.* Genome-wide association study of paliperidone efficacy. *Pharmacogenetics and genomics* 2016.

26. Wray NR, Ripke S, Mattheisen M, Trzaskowski M, Byrne EM, Abdellaoui A *et al.* Genome-wide association analyses identify 44 risk variants and refine the genetic architecture of major depression. *Nature genetics* 2018.

27. Bigdeli TB, Ripke S, Peterson RE, Trzaskowski M, Bacanu SA, Abdellaoui A *et al.* Genetic effects influencing risk for major depressive disorder in China and Europe. *Translational psychiatry* 2017; **7**(3)**:** e1074.

28. Witt SH, Streit F, Jungkunz M, Frank J, Awasthi S, Reinbold CS *et al.* Genome-wide association study of borderline personality disorder reveals genetic overlap with bipolar disorder, major depression and schizophrenia. *Translational psychiatry* 2017; **7**(6)**:** e1155.

29. Li QS, Wajs E, Ochs-Ross R, Singh J, Drevets WC. Genome-wide association study and polygenic risk score analysis of esketamine treatment response. *Scientific reports* 2020; **10**(1)**:** 12649.

30. Stahl EA, Breen G, Forstner AJ, McQuillin A, Ripke S, Trubetskoy V *et al.* Genome-wide association study identifies 30 loci associated with bipolar disorder. *Nature genetics* 2019; **51**(5)**:** 793-803.

31. Mullins N, Forstner AJ, O'Connell KS, Coombes B, Coleman JRI, Qiao Z *et al.* Genome-wide association study of more than 40,000 bipolar disorder cases provides new insights into the underlying biology. *Nature genetics* 2021.

32. Chang CC, Chow CC, Tellier LC, Vattikuti S, Purcell SM, Lee JJ. Second-generation PLINK: rising to the challenge of larger and richer datasets. *Gigascience* 2015; **4:** 7.

33. Lam M, Awasthi S, Watson HJ, Goldstein J, Panagiotaropoulou G, Trubetskoy V *et al.* RICOPILI: Rapid Imputation for COnsortias PIpeLIne. *Bioinformatics* 2020; **36**(3)**:** 930-933.

34. Patterson N, Price AL, Reich D. Population structure and eigenanalysis. *PLoS genetics* 2006; **2**(12)**:** e190.

35. Price AL, Patterson NJ, Plenge RM, Weinblatt ME, Shadick NA, Reich D. Principal components analysis corrects for stratification in genome-wide association studies. *Nature genetics* 2006; **38**(8)**:** 904-909.

36. Das S, Forer L, Schonherr S, Sidore C, Locke AE, Kwong A *et al.* Next-generation genotype imputation service and methods. *Nature genetics* 2016; **48**(10)**:** 1284-1287.

37. Loh PR, Danecek P, Palamara PF, Fuchsberger C, Y AR, H KF *et al.* Reference-based phasing using the Haplotype Reference Consortium panel. *Nature genetics* 2016; **48**(11)**:** 1443-1448.

38. Mullins N, Bigdeli TB, Borglum AD, Coleman JRI, Demontis D, Mehta D *et al.* GWAS of Suicide Attempt in Psychiatric Disorders and Association With Major Depression Polygenic Risk Scores. *The American journal of psychiatry* 2019; **176**(8)**:** 651-660.

39. Howard DM, Adams MJ, Clarke TK, Hafferty JD, Gibson J, Shirali M *et al.* Genome-wide meta-analysis of depression identifies 102 independent variants and highlights the importance of the prefrontal brain regions. *Nature neuroscience* 2019; **22**(3)**:** 343-352.

40. Nagel M, Jansen PR, Stringer S, Watanabe K, de Leeuw CA, Bryois J *et al.* Meta-analysis of genome-wide association studies for neuroticism in 449,484 individuals identifies novel genetic loci and pathways. *Nature genetics* 2018; **50**(7)**:** 920-927.

41. Demontis D, Walters RK, Martin J, Mattheisen M, Als TD, Agerbo E *et al.* Discovery of the first genome-wide significant risk loci for attention deficit/hyperactivity disorder. *Nature genetics* 2019; **51**(1)**:** 63-75.

42. Grove J, Ripke S, Als TD, Mattheisen M, Walters RK, Won H *et al.* Identification of common genetic risk variants for autism spectrum disorder. *Nature genetics* 2019; **51**(3)**:** 431-444.

43. Nievergelt CM, Maihofer AX, Klengel T, Atkinson EG, Chen CY, Choi KW *et al.* International meta-analysis of PTSD genome-wide association studies identifies sex- and ancestry-specific genetic risk loci. *Nature communications* 2019; **10**(1)**:** 4558.

44. Otowa T, Hek K, Lee M, Byrne EM, Mirza SS, Nivard MG *et al.* Meta-analysis of genome-wide association studies of anxiety disorders. *Molecular psychiatry* 2016; **21**(10)**:** 1485.

45. Meier SM, Trontti K, Purves KL, Als TD, Grove J, Laine M *et al.* Genetic Variants Associated With Anxiety and Stress-Related Disorders: A Genome-Wide Association Study and Mouse-Model Study. *JAMA Psychiatry* 2019; **76**(9)**:** 924-932.

46. Johnson EC, Demontis D, Thorgeirsson TE, Walters RK, Polimanti R, Hatoum AS *et al.* A large-scale genome-wide association study meta-analysis of cannabis use disorder. *Lancet Psychiatry* 2020; **7**(12)**:** 1032-1045.

47. Liu M, Jiang Y, Wedow R, Li Y, Brazel DM, Chen F *et al.* Association studies of up to 1.2 million individuals yield new insights into the genetic etiology of tobacco and alcohol use. *Nature genetics* 2019; **51**(2)**:** 237-244.

48. Lee JJ, Wedow R, Okbay A, Kong E, Maghzian O, Zacher M *et al.* Gene discovery and polygenic prediction from a genome-wide association study of educational attainment in 1.1 million individuals. *Nature genetics* 2018; **50**(8)**:** 1112-1121.

49. Savage JE, Jansen PR, Stringer S, Watanabe K, Bryois J, de Leeuw CA *et al.* Genome-wide association meta-analysis in 269,867 individuals identifies new genetic and functional links to intelligence. *Nature genetics* 2018; **50**(7)**:** 912-919.

50. Davies G, Lam M, Harris SE, Trampush JW, Luciano M, Hill WD *et al.* Study of 300,486 individuals identifies 148 independent genetic loci influencing general cognitive function. *Nature communications* 2018; **9**(1)**:** 2098.

51. Jansen IE, Savage JE, Watanabe K, Bryois J, Williams DM, Steinberg S *et al.* Genome-wide meta-analysis identifies new loci and functional pathways influencing Alzheimer's disease risk. *Nature genetics* 2019; **51**(3)**:** 404-413.

52. Johnston KJA, Adams MJ, Nicholl BI, Ward J, Strawbridge RJ, Ferguson A *et al.* Genome-wide association study of multisite chronic pain in UK Biobank. *PLoS genetics* 2019; **15**(6)**:** e1008164.

53. Suri P, Palmer MR, Tsepilov YA, Freidin MB, Boer CG, Yau MS *et al.* Genome-wide meta-analysis of 158,000 individuals of European ancestry identifies three loci associated with chronic back pain. *PLoS genetics* 2018; **14**(9)**:** e1007601.

54. Okbay A, Baselmans BM, De Neve JE, Turley P, Nivard MG, Fontana MA *et al.* Genetic variants associated with subjective well-being, depressive symptoms, and neuroticism identified through genome-wide analyses. *Nature genetics* 2016; **48**(6)**:** 624-633.

55. Hibar DP, Stein JL, Renteria ME, Arias-Vasquez A, Desrivieres S, Jahanshad N *et al.* Common genetic variants influence human subcortical brain structures. *Nature* 2015; **520**(7546)**:** 224-229.

56. Karlsson Linner R, Biroli P, Kong E, Meddens SFW, Wedow R, Fontana MA *et al.* Genome-wide association analyses of risk tolerance and risky behaviors in over 1 million individuals identify hundreds of loci and shared genetic influences. *Nature genetics* 2019; **51**(2)**:** 245-257.

57. Ligthart S, Vaez A, Vosa U, Stathopoulou MG, de Vries PS, Prins BP *et al.* Genome Analyses of >200,000 Individuals Identify 58 Loci for Chronic Inflammation and Highlight Pathways that Link Inflammation and Complex Disorders. *American journal of human genetics* 2018; **103**(5)**:** 691-706.

58. Teslovich TM, Musunuru K, Smith AV, Edmondson AC, Stylianou IM, Koseki M *et al.* Biological, clinical and population relevance of 95 loci for blood lipids. *Nature* 2010; **466**(7307)**:** 707-713.

59. Willer CJ, Schmidt EM, Sengupta S, Peloso GM, Gustafsson S, Kanoni S *et al.* Discovery and refinement of loci associated with lipid levels. *Nature genetics* 2013; **45**(11)**:** 1274-1283.

60. van der Harst P, Verweij N. Identification of 64 Novel Genetic Loci Provides an Expanded View on the Genetic Architecture of Coronary Artery Disease. *Circ Res* 2018; **122**(3)**:** 433-443.

61. Shungin D, Winkler TW, Croteau-Chonka DC, Ferreira T, Locke AE, Magi R *et al.* New genetic loci link adipose and insulin biology to body fat distribution. *Nature* 2015; **518**(7538)**:** 187-196.

62. Yengo L, Sidorenko J, Kemper KE, Zheng Z, Wood AR, Weedon MN *et al.* Meta-analysis of genome-wide association studies for height and body mass index in approximately 700000 individuals of European ancestry. *Human molecular genetics* 2018; **27**(20)**:** 3641-3649.

63. Forstner AJ, Awasthi S, Wolf C, Maron E, Erhardt A, Czamara D *et al.* Genome-wide association study of panic disorder reveals genetic overlap with neuroticism and depression. *Molecular psychiatry* 2021; **26**(8)**:** 4179-4190.

64. Strawbridge RJ, Ward J, Ferguson A, Graham N, Shaw RJ, Cullen B *et al.* Identification of novel genome-wide associations for suicidality in UK Biobank, genetic correlation with psychiatric disorders and polygenic association with completed suicide. *EBioMedicine* 2019; **41:** 517-525.

65. Li M, Li Y, Qin H, Tubbs JD, Li M, Qiao C *et al.* Genome-wide DNA methylation analysis of peripheral blood cells derived from patients with first-episode schizophrenia in the Chinese Han population. *Molecular psychiatry* 2021; **26**(8)**:** 4475-4485.

66. Datta D, Arion D, Corradi JP, Lewis DA. Altered expression of CDC42 signaling pathway components in cortical layer 3 pyramidal cells in schizophrenia. *Biological psychiatry* 2015; **78**(11)**:** 775-785.

67. Hirayama-Kurogi M, Takizawa Y, Kunii Y, Matsumoto J, Wada A, Hino M *et al.* Downregulation of GNA13-ERK network in prefrontal cortex of schizophrenia brain identified by combined focused and targeted quantitative proteomics. *J Proteomics* 2017; **158:** 31-42.

68. Bethea CL, Reddy AP. Effect of ovarian hormones on genes promoting dendritic spines in laser-captured serotonin neurons from macaques. *Molecular psychiatry* 2010; **15**(10)**:** 1034-1044.

69. Toma C, Shaw AD, Heath A, Pierce KD, Mitchell PB, Schofield PR *et al.* A linkage and exome study of multiplex families with bipolar disorder implicates rare coding variants of ANK3 and additional rare alleles at 10q11-q21. *J Psychiatry Neurosci* 2021; **46**(2)**:** E247-E257.
